# Pure Hereditary Spastic Paraplegia in a Patient With a Novel Heterozygous KIDINS220 Gene Mutation

**DOI:** 10.7759/cureus.64023

**Published:** 2024-07-07

**Authors:** Hassan S Al Hussein, Lauren M Guerra, Syed Ali Raza, Vijaykumar Javalkar, Madiha Raza

**Affiliations:** 1 Department of Neurology (Medicine), Hamad General Hospital, Doha, QAT; 2 Neuroscience, Northwestern University, Evanston, USA; 3 Neurology, National Institutes of Health, Bethesda, USA; 4 Neurology, Ochsner LSU (Louisiana State University) Health Shreveport - Academic Medical Center, Shreveport, USA; 5 Neurology, Ziauddin University, Karachi, PAK

**Keywords:** sino syndrome, kidins220, sanger sequencing, next-generation sequencing (ngs), hereditary spastic paraplegia (hsp)

## Abstract

This case presents a somewhat unique and different phenotype of hereditary spastic paraplegia from previously reported kinase D-interacting substrate of 220 kDa (*KIDINS220*) gene mutation-related disease. We report a unique putative causative heterozygous mutation in *KIDINS220* in a pure hereditary spastic paraplegia (HSP) patient expanding the HSP group further. We also deliberate on how our case was different from prior *KIDINS220*-related pathologies including spastic paraplegia, intellectual disability, nystagmus, and obesity (SINO) syndrome, and the observation of *KIDINS220* and aquaporin-4 (*AQP4*) downregulation in the ventricular ependymal lining of idiopathic normal pressure hydrocephalus (iNPH) patients. These findings warrant further investigations of the biology of *KIDINS220*. With the advent of new gene editing technologies like Clustered Regularly Interspaced Short Palindromic Repeats (CRISPR)/CRISPR-associated protein 9 (Cas9), variants such as ours provide an opportunity for targeted precision medicine.

## Introduction

Hereditary spastic paraplegia (HSP) is a heterogeneous group of genetic neurodegenerative disorders characterized by spasticity and weakness in the lower limbs, stemming from degeneration of the corticospinal tract. HSP is categorized into two main types: (i) Pure HSP, which primarily involves spastic paraparesis with minimal sensory symptoms, and (ii) Complex HSP which includes additional neurological and non-neurological features [1].

The inheritance patterns can be autosomal dominant, autosomal recessive, or X-linked, with sporadic cases accounting for 13-40% of all reported cases [2]. Kinase D-interacting substrate of 220 kDa (*KIDINS220*) encodes a conserved membrane protein that has 11 ankyrin repeats and four transmembrane domains [3]. It is a scaffold protein involved in signal transduction, thereby playing crucial roles in neuronal cell survival, synaptic plasticity, and B and T cell activation and development [4]. Apart from its role in neurotrophic pathways in neurons, it was also shown to be required for the establishment of proper neuronal connectivity and in astrocyte-neuron communication [5].

Previously de novo nonsense variants in *KIDINS220* were observed in patients with spastic paraplegia, intellectual disability, nystagmus, and obesity (SINO) syndrome [6]. Recently, KIDINS220-sorting nexin 27 (SNX27)-retromer pathway was also demonstrated to be regulatory for brain aquaporin-4 (AQP4) expression with KIDINS220 deficiency resulting in AQP4 lysosomal degradation [7]. Expression of KIDINS220 and AQP4 was found to be decreased in the ventricular ependymal lining of patients with idiopathic normal pressure hydrocephalus (iNPH) [7].

## Case presentation

A 23-year-old Caucasian female presented with long-standing progressive gait difficulty and lower extremity stiffness that began to occur seven years prior to this presentation. At that time, extensive work-up was performed which was negative including magnetic resonance imaging (MRI) of the entire neuroaxis. No genetic testing was performed since there was no family history of gait abnormality reported. At this presentation, the patient also reported mild dysarthria, slight difficulty performing fine motor movements of the upper extremities likely attributable to very minimal coordination deficits, and urinary urgency, with otherwise no sphincter dysfunction. Her medical history was unremarkable; she had a normal birth and no developmental delay. Her family history was unremarkable as well. The patient did not have any psychiatric conditions.

The general examination was unremarkable and there were no deformities - she had no history of developmental anomalies or any cognitive delay. Her neurological examination demonstrated mild dysarthria, bilateral upper extremities’ postural tremor, spasticity of the lower extremities (Modified Ashworth Scale 2), exaggerated knee (+3) and ankle (4+) deep tendon reflexes with sustained bilateral ankle clonus and bilateral up-going plantar reflexes. At the time of the visit, she was cognitively intact with good recall, intact attention, and comprehension. She had a normal cranial nerve exam and no concerns were raised for diplopia. Her visual acuity testing and visual field testing were also negative. Her ophthalmologic exam did not reveal any optic disc abnormalities and she had no visual complaints. The sensory exam was normal for all modalities. She had normal tone and reflexes in the upper extremities, and no cerebellar signs were noted.

Work-up for non-structural causes including routine blood tests, thyroid-stimulating hormone (TSH), vitamin B12, copper serum level, ceruloplasmin levels, vitamin E level, HIV, rapid plasma reagin (RPR), homocysteine and plasma very long chain fatty acids were negative. Liver function tests and lipid panel were also unremarkable. We also performed basic cerebrospinal fluid (CSF) testing which was unremarkable. CSF testing for the autoimmune panel and infectious etiologies were also negative. MRI of the brain and spine with and without contrast was repeated and was unremarkable again. Since the workup for compressive and non-compressive myelopathy was unremarkable and the examination findings of spasticity in lower extremities sparing the upper extremities were present, HSP was suspected, and a genetic panel was sent following genetic counseling.

Next-generation sequencing (NGS) and Sanger sequencing discovered c.2183A>T (p.Asn728Ile) missense variant in *KIDINS220*. The variant was absent in controls (i.e. gnomAD and Exome Sequencing Project) (PM2 criteria). Note that in silico prediction programs supported an uncertain significance of the variant (using VarSome, Lausanne, Vaud) [8]. However, the patient’s phenotype was highly suggestive of HSP with a monogenic etiology (PP4 criteria). We curated the Exome variant list for *KIDINS220* from the exome sequencing project (see Appendices). For the variant c.2185G>A (p.A729T), the Polyphen2 (class: score) is listed as probably-damaging: 0.997 (in bold in the table in Appendices). Since this was adjacent to the variant found in our patient (c.2183A>T (p.N728I)), one would expect the variant in our patient to be probably pathogenic as well. Based on the abovementioned American College of Medical Genetics criteria [9] and caveats, one could suggest the likely pathogenicity of this variant. The other mutation discovered (CYP27A1 (c.546T>G, p.Ile182Met)) would not have fit the phenotypic description of the patient, and considering variant allele frequency, it was less likely to be contributive. Note though, although it seemed plausible that the case was sporadic, we were unable to perform NGS on the family members to provide clear evidence for it.

Initially, the patient's symptoms were managed with physical and occupational therapy. Baclofen was also used for spasticity. She was neurologically stable during a close follow-up visit; however, it was felt that she would require further titration of antispasmodics for the lower extremity spasticity.

## Discussion

HSP is a heterogeneous group of monogenic progressive neurodegenerative disorders characterized by the involvement of corticospinal tract and dorsal columns [10]. The prototypical presentation in the pure type involves slowly progressive spastic paraparesis, urinary incontinence, and minimal sensory symptoms. In the complex type, other nervous system manifestations including cognitive impairment, extrapyramidal involvement, peripheral neuropathy, optic neuropathy, retinal disease, seizures, and more pronounced sensory symptoms may be present. The genetic classification of HSP is based on the spastic paraplegia gene (SPG) designation. Table 1 characterizes HSP based on the mode of inheritance with some common subtypes highlighted; there is heterogeneity in terms of gene involvement and clinical presentation [10].

**Table 1:** HSP classified based on modes of inheritance. Note that this is by no means a complete list of HSP subtypes References: [10,11] HSP: hereditary spastic paraplegia; AR: autosomal recessive; AD: autosomal dominant; SPG: spastic paraplegia gene

Mode of inheritance	Subtypes	Clinical and radiographic features
AD HSP	SPG4: most common AD HSP; associated with mutations in *SPAST*	Classical disease - pure phenotype
	SPG3A: second most common AD HSP; associated with mutations in *ATL1*	Pure phenotype, seizures, thin corpus callosum, rarely optic atrophy
	SPG31: third most common AD HSP; associated with mutations in *REEP1*	Usually presents as pure phenotype, but is associated with axonal neuropathy in approximately ~ 50% of cases
AR HSP	SPG11: one of the most prevalent AR HSPs; mutations in *SPG11*	‘Ear of the lynx sign’, cases can present with cognitive impairment; lower extremity spasticity, ataxia, dysarthria, axonal motor neuropathy, parkinsonism, optic atrophy
	SPG15: associated with mutations in *ZFYVE26*	Thin corpus callosum, lower extremity spasticity levodopa-responsive parkinsonism, axonal neuropathy, adducted thumbs
	SPG5: mutations in *CYP7B1*	Sensory ataxia, dorsal column dysfunction
	SPG7: mutations in *SPG7*	Ataxia, external ophthalmoplegia, proximal myopathic weakness, lower extremity spasticity
X-linked HSP	SPG2: mutations in *PLP1*	Variant of variant of Pelizaeus-Merzbacher disease- can have diffuse hypomyelination pattern

Our patient was unique in having primarily pure HSP features associated with the *KIDINS220* missense variant reported (c.2183A>T(p.Asn728Ile)) here which expands the HSP spectrum further. This is different from the prior *KIDINS220* nonsense variants leading to SINO [6]. Although we suggest likely pathogenicity of our variant, confirmation of pathogenicity would entail functional studies. One possible approach would be to induce our missense *KIDINS220* variant in motor neurons (using Gal4/UAS) to look for spasms in zebrafish as performed previously [6]. Additionally, our patient did not have any features of ventriculomegaly or iNPH as noted above [7]. This warrants further investigation of the biology of* KIDINS220*.

## Conclusions

We described a case of pure HSP with a likely pathogenic *KIDINS220* variant. This case helps expand the HSP subtype spectrum and adds to the growing body of knowledge regarding the genetic heterogeneity of this condition. This provides opportunities for greater pathophysiological understanding and targeted molecular therapy.

## Appendices

**Table 2:** Exome variant list curation for KIDINS220 from exome sequencing project References: [9,12,13]

#base (NCBI.37)	rsID	dbSNP Version	Alleles	European American Allele Count	African American Allele Count	All Allele Count	MAFinPercent(EA/AA/All)	European American Genotype Count	African American Genotype Count	All Genotype Count	Average Sample Read Depth	Genes	Gene Accession	FunctionGVS	hgvsProteinVariant	hgvsCdnaVariant	codingDnaSize	ConservationScorePhastCons	ConservationScoreGERP	GranthamScore	Polyphen2(Class:Score)	RefBaseNCBI37	ChimpAllele	ClinicalInfo	FilterStatus	OnIlluminaHumanExomeChip	GwasPubMedInfo	EA-EstimatedAge(kyrs)	AA-EstimatedAge(kyrs)	GRCh38Position
2:8870814	rs6431959	dbSNP_116	A>G	G=8205/A=95	G=3386/A=512	G=11591/A=607	1.1446/13.1349/4.9762	GG=4057/GA=91/AA=2	GG=1476/GA=434/AA=39	GG=5533/GA=525/AA=41	34	KIDINS220	NM_020738.2	utr-3	NA	c.*36T>C	5316	0	0.21	NA	unknown	A	G	unknown	PASS	no	unknown	NA	NA	2:8730684
2:8870862	rs13406422	dbSNP_121	T>C	C=22/T=8220	C=10/T=3780	C=32/T=12000	0.2669/0.2639/0.266	CC=0/CT=22/TT=4099	CC=0/CT=10/TT=1885	CC=0/CT=32/TT=5984	71	KIDINS220	NM_020738.2	coding-synonymous	p.(E1768=)	c.5304A>G	5316	0.987	-1.57	NA	unknown	T	T	unknown	PASS	no	unknown	NA	NA	2:8730732
2:8870959	rs375496239	dbSNP_138	C>T	T=2/C=8244	T=0/C=3824	T=2/C=12068	0.0243/0.0/0.0166	TT=0/TC=2/CC=4121	TT=0/TC=0/CC=1912	TT=0/TC=2/CC=6033	156	KIDINS220	NM_020738.2	missense	p.(R1736Q)	c.5207G>A	5316	0.985	5.93	43	probably-damaging:1.0	C	C	unknown	PASS	no	unknown	NA	NA	2:8730829
2:8871015	rs368542023	dbSNP_138	G>A	A=1/G=8249	A=0/G=3816	A=1/G=12065	0.0121/0.0/0.0083	AA=0/AG=1/GG=4124	AA=0/AG=0/GG=1908	AA=0/AG=1/GG=6032	153	KIDINS220	NM_020738.2	coding-synonymous	p.(S1717=)	c.5151C>T	5316	0.197	3.15	NA	unknown	G	G	unknown	PASS	no	unknown	NA	NA	2:8730885
2:8871029	rs372797696	dbSNP_138	A>G	G=1/A=8243	G=0/A=3798	G=1/A=12041	0.0121/0.0/0.0083	GG=0/GA=1/AA=4121	GG=0/GA=0/AA=1899	GG=0/GA=1/AA=6020	149	KIDINS220	NM_020738.2	coding-synonymous	p.(L1713=)	c.5137T>C	5316	0	-10.8	NA	unknown	A	A	unknown	PASS	no	unknown	NA	NA	2:8730899
2:8871074	rs373610139	dbSNP_138	T>C	C=3/T=8261	C=1/T=3809	C=4/T=12070	0.0363/0.0262/0.0331	CC=0/CT=3/TT=4129	CC=0/CT=1/TT=1904	CC=0/CT=4/TT=6033	116	KIDINS220	NM_020738.2	missense	p.(N1698D)	c.5092A>G	5316	0.007	2.15	23	possibly-damaging:0.604	T	T	unknown	PASS	no	unknown	NA	NA	2:8730944
2:8871119	rs367740187	dbSNP_138	C>T	T=1/C=8305	T=0/C=3896	T=1/C=12201	0.012/0.0/0.0082	TT=0/TC=1/CC=4152	TT=0/TC=0/CC=1948	TT=0/TC=1/CC=6100	104	KIDINS220	NM_020738.2	missense	p.(V1683M)	c.5047G>A	5316	0.294	3.19	21	benign:0.41	C	C	unknown	PASS	no	unknown	NA	NA	2:8730989
2:8871164	rs371994059	dbSNP_138	G>A	A=0/G=8298	A=1/G=3875	A=1/G=12173	0.0/0.0258/0.0082	AA=0/AG=0/GG=4149	AA=0/AG=1/GG=1937	AA=0/AG=1/GG=6086	90	KIDINS220	NM_020738.2	missense	p.(P1668S)	c.5002C>T	5316	0.999	5.93	74	probably-damaging:1.0	G	G	unknown	PASS	no	unknown	NA	NA	2:8731034
2:8871191	rs376904754	dbSNP_138	T>C	C=0/T=8266	C=1/T=3877	C=1/T=12143	0.0/0.0258/0.0082	CC=0/CT=0/TT=4133	CC=0/CT=1/TT=1938	CC=0/CT=1/TT=6071	84	KIDINS220	NM_020738.2	missense	p.(I1659V)	c.4975A>G	5316	0.868	2.09	29	benign:0.121	T	T	unknown	PASS	no	unknown	NA	NA	2:8731061
2:8871204	rs369561699	dbSNP_138	G>A	A=0/G=8248	A=1/G=3863	A=1/G=12111	0.0/0.0259/0.0083	AA=0/AG=0/GG=4124	AA=0/AG=1/GG=1931	AA=0/AG=1/GG=6055	81	KIDINS220	NM_020738.2	coding-synonymous	p.(S1654=)	c.4962C>T	5316	0.01	-12.1	NA	unknown	G	G	unknown	PASS	no	unknown	NA	NA	2:8731074
2:8871219	rs202094672	dbSNP_137	G>C	C=0/G=8266	C=20/G=3848	C=20/G=12114	0.0/0.5171/0.1648	CC=0/CG=0/GG=4133	CC=0/CG=20/GG=1914	CC=0/CG=20/GG=6047	81	KIDINS220	NM_020738.2	missense	p.(D1649E)	c.4947C>G	5316	1	3.24	45	probably-damaging:1.0	G	G	unknown	PASS	yes	unknown	NA	NA	2:8731089
2:8871251	rs376497503	dbSNP_138	T>C	C=1/T=8265	C=0/T=3894	C=1/T=12159	0.0121/0.0/0.0082	CC=0/CT=1/TT=4132	CC=0/CT=0/TT=1947	CC=0/CT=1/TT=6079	77	KIDINS220	NM_020738.2	missense	p.(I1639V)	c.4915A>G	5316	0	-11.8	29	benign:0.0	T	T	unknown	PASS	no	unknown	NA	NA	2:8731121
2:8871258	rs373974134	dbSNP_138	T>C	C=1/T=8279	C=0/T=3914	C=1/T=12193	0.0121/0.0/0.0082	CC=0/CT=1/TT=4139	CC=0/CT=0/TT=1957	CC=0/CT=1/TT=6096	75	KIDINS220	NM_020738.2	coding-synonymous	p.(Q1636=)	c.4908A>G	5316	0.005	-5.48	NA	unknown	T	T	unknown	PASS	no	unknown	NA	NA	2:8731128
2:8871272	rs200409469	dbSNP_137	G>C	C=2/G=8290	C=0/G=3954	C=2/G=12244	0.0241/0.0/0.0163	CC=0/CG=2/GG=4144	CC=0/CG=0/GG=1977	CC=0/CG=2/GG=6121	74	KIDINS220	NM_020738.2	missense	p.(L1632V)	c.4894C>G	5316	0.112	-9.87	32	probably-damaging:0.999	G	G	unknown	PASS	yes	unknown	NA	NA	2:8731142
2:8871287	rs369024354	dbSNP_138	C>T	T=0/C=8316	T=1/C=3967	T=1/C=12283	0.0/0.0252/0.0081	TT=0/TC=0/CC=4158	TT=0/TC=1/CC=1983	TT=0/TC=1/CC=6141	73	KIDINS220	NM_020738.2	missense	p.(G1627R)	c.4879G>A	5316	0.372	5.92	125	probably-damaging:1.0	C	C	unknown	PASS	no	unknown	NA	NA	2:8731157
2:8871302	rs372833860	dbSNP_138	G>A	A=0/G=8336	A=2/G=4000	A=2/G=12336	0.0/0.05/0.0162	AA=0/AG=0/GG=4168	AA=0/AG=2/GG=1999	AA=0/AG=2/GG=6167	71	KIDINS220	NM_020738.2	missense	p.(H1622Y)	c.4864C>T	5316	0.019	5.92	83	possibly-damaging:0.881	G	G	unknown	PASS	no	unknown	NA	NA	2:8731172
2:8871317	rs376087742	dbSNP_138	G>T	T=0/G=8366	T=1/G=4045	T=1/G=12411	0.0/0.0247/0.0081	TT=0/TG=0/GG=4183	TT=0/TG=1/GG=2022	TT=0/TG=1/GG=6205	69	KIDINS220	NM_020738.2	missense	p.(L1617M)	c.4849C>A	5316	0.993	5.05	15	probably-damaging:1.0	G	G	unknown	PASS	no	unknown	NA	NA	2:8731187
2:8871342	rs1044280	dbSNP_86	C>A	A=2209/C=6181	A=883/C=3163	A=3092/C=9344	26.329/21.824/24.8633	AA=288/AC=1633/CC=2274	AA=93/AC=697/CC=1233	AA=381/AC=2330/CC=3507	69	KIDINS220	NM_020738.2	missense	p.(Q1608H)	c.4824G>T	5316	0.993	2.88	24	benign:0.002	C	C	unknown	PASS	yes	unknown	NA	NA	2:8731212
2:8871353	rs374139570	dbSNP_138	C>T	T=1/C=8391	T=0/C=4076	T=1/C=12467	0.0119/0.0/0.008	TT=0/TC=1/CC=4195	TT=0/TC=0/CC=2038	TT=0/TC=1/CC=6233	73	KIDINS220	NM_020738.2	missense	p.(D1605N)	c.4813G>A	5316	0.036	5.92	23	probably-damaging:0.968	C	C	unknown	PASS	no	unknown	NA	NA	2:8731223
2:8871372	rs377371016	dbSNP_138	A>G	G=1/A=8393	G=0/A=4086	G=1/A=12479	0.0119/0.0/0.008	GG=0/GA=1/AA=4196	GG=0/GA=0/AA=2043	GG=0/GA=1/AA=6239	89	KIDINS220	NM_020738.2	coding-synonymous	p.(S1598=)	c.4794T>C	5316	0.462	-8.76	NA	unknown	A	A	unknown	PASS	no	unknown	NA	NA	2:8731242
2:8871439	rs202036173	dbSNP_137	G>A	A=1/G=8347	A=0/G=3956	A=1/G=12303	0.012/0.0/0.0081	AA=0/AG=1/GG=4173	AA=0/AG=0/GG=1978	AA=0/AG=1/GG=6151	129	KIDINS220	NM_020738.2	missense	p.(A1576V)	c.4727C>T	5316	0.388	5.92	64	probably-damaging:1.0	G	G	unknown	PASS	yes	unknown	NA	NA	2:8731309
2:8871509	rs375631503	dbSNP_138	G>A	A=2/G=8304	A=0/G=3928	A=2/G=12232	0.0241/0.0/0.0163	AA=0/AG=2/GG=4151	AA=0/AG=0/GG=1964	AA=0/AG=2/GG=6115	133	KIDINS220	NM_020738.2	missense	p.(P1553S)	c.4657C>T	5316	0	-4.69	74	benign:0.0	G	G	unknown	PASS	no	unknown	NA	NA	2:8731379
2:8871555	rs367620717	dbSNP_138	G>C	C=2/G=8292	C=0/G=3846	C=2/G=12138	0.0241/0.0/0.0165	CC=0/CG=2/GG=4145	CC=0/CG=0/GG=1923	CC=0/CG=2/GG=6068	91	KIDINS220	NM_020738.2	coding-synonymous	p.(L1537=)	c.4611C>G	5316	0.422	1.57	NA	unknown	G	G	unknown	PASS	no	unknown	NA	NA	2:8731425
2:8871615	rs200002699	dbSNP_137	T>C	C=2/T=8234	C=0/T=3816	C=2/T=12050	0.0243/0.0/0.0166	CC=0/CT=2/TT=4116	CC=0/CT=0/TT=1908	CC=0/CT=2/TT=6024	62	KIDINS220	NM_020738.2	coding-synonymous	p.(Q1517=)	c.4551A>G	5316	0.645	-3.04	NA	unknown	T	T	unknown	PASS	yes	unknown	NA	NA	2:8731485
2:8871622	rs375231110	dbSNP_138	C>T	T=0/C=8234	T=1/C=3811	T=1/C=12045	0.0/0.0262/0.0083	TT=0/TC=0/CC=4117	TT=0/TC=1/CC=1905	TT=0/TC=1/CC=6022	60	KIDINS220	NM_020738.2	missense	p.(R1515H)	c.4544G>A	5316	1	5.92	29	probably-damaging:1.0	C	C	unknown	PASS	no	unknown	NA	NA	2:8731492
2:8871716	rs367866337	dbSNP_138	C>G	G=2/C=8210	G=0/C=3774	G=2/C=11984	0.0244/0.0/0.0167	GG=0/GC=2/CC=4104	GG=0/GC=0/CC=1887	GG=0/GC=2/CC=5991	66	KIDINS220	NM_020738.2	missense	p.(D1484H)	c.4450G>C	5316	0.384	5.92	81	probably-damaging:0.999	C	C	unknown	PASS	no	unknown	NA	NA	2:8731586
2:8871797	rs201478192	dbSNP_137	C>A	A=3/C=8195	A=1/C=3781	A=4/C=11976	0.0366/0.0264/0.0334	AA=0/AC=3/CC=4096	AA=0/AC=1/CC=1890	AA=0/AC=4/CC=5986	131	KIDINS220	NM_020738.2	missense	p.(V1457F)	c.4369G>T	5316	0.978	5.92	50	probably-damaging:1.0	C	C	unknown	PASS	yes	unknown	NA	NA	2:8731667
2:8871897	rs375458262	dbSNP_138	T>C	C=0/T=8196	C=1/T=3703	C=1/T=11899	0.0/0.027/0.0084	CC=0/CT=0/TT=4098	CC=0/CT=1/TT=1851	CC=0/CT=1/TT=5949	135	KIDINS220	NM_020738.2	coding-synonymous	p.(S1423=)	c.4269A>G	5316	0.129	-7.12	NA	unknown	T	C	unknown	PASS	no	unknown	NA	NA	2:8731767
2:8871904	rs369897192	dbSNP_138	C>T	T=1/C=8203	T=0/C=3706	T=1/C=11909	0.0122/0.0/0.0084	TT=0/TC=1/CC=4101	TT=0/TC=0/CC=1853	TT=0/TC=1/CC=5954	132	KIDINS220	NM_020738.2	missense	p.(S1421N)	c.4262G>A	5316	0.977	5.87	46	probably-damaging:0.999	C	C	unknown	PASS	no	unknown	NA	NA	2:8731774
2:8871911	rs372241002	dbSNP_138	C>G	G=1/C=8205	G=0/C=3702	G=1/C=11907	0.0122/0.0/0.0084	GG=0/GC=1/CC=4102	GG=0/GC=0/CC=1851	GG=0/GC=1/CC=5953	130	KIDINS220	NM_020738.2	missense	p.(G1419R)	c.4255G>C	5316	0.991	5.87	125	probably-damaging:1.0	C	C	unknown	PASS	no	unknown	NA	NA	2:8731781
2:8871963	rs183360862	dbSNP_135	G>A	A=0/G=8194	A=1/G=3673	A=1/G=11867	0.0/0.0272/0.0084	AA=0/AG=0/GG=4097	AA=0/AG=1/GG=1836	AA=0/AG=1/GG=5933	112	KIDINS220	NM_020738.2	coding-synonymous	p.(P1401=)	c.4203C>T	5316	0.006	-8.26	NA	unknown	G	G	unknown	PASS	no	unknown	NA	NA	2:8731833
2:8871973	rs368915698	dbSNP_138	T>C	C=0/T=8198	C=1/T=3669	C=1/T=11867	0.0/0.0272/0.0084	CC=0/CT=0/TT=4099	CC=0/CT=1/TT=1834	CC=0/CT=1/TT=5933	112	KIDINS220	NM_020738.2	missense	p.(E1398G)	c.4193A>G	5316	1	5.86	98	probably-damaging:1.0	T	T	unknown	PASS	no	unknown	NA	NA	2:8731843
2:8871982	rs372410720	dbSNP_138	G>A	A=0/G=8202	A=1/G=3693	A=1/G=11895	0.0/0.0271/0.0084	AA=0/AG=0/GG=4101	AA=0/AG=1/GG=1846	AA=0/AG=1/GG=5947	111	KIDINS220	NM_020738.2	missense	p.(S1395F)	c.4184C>T	5316	0.993	5.86	155	probably-damaging:1.0	G	G	unknown	PASS	no	unknown	NA	NA	2:8731852
2:8872132	unknown	none	AT>A	A1=12/R=7770	A1=1/R=3463	A1=13/R=11233	0.1542/0.0289/0.1156	A1A1=0/A1R=12/RR=3879	A1A1=0/A1R=1/RR=1731	A1A1=0/A1R=13/RR=5610	28	KIDINS220	NM_020738.2	intron	NA	c.4054-21del1	5316	0.877	5.68	unknown	unknown	AT	unknown	unknown	PASS	no	unknown	NA	NA	2:8732002
2:8872136	rs374753023	dbSNP_138	T>C	C=1/T=8107	C=0/T=3570	C=1/T=11677	0.0123/0.0/0.0086	CC=0/CT=1/TT=4053	CC=0/CT=0/TT=1785	CC=0/CT=1/TT=5838	25	KIDINS220	NM_020738.2	intron	NA	c.4054-24A>G	5316	0.274	0.23	NA	unknown	T	T	unknown	PASS	no	unknown	NA	NA	2:8732006
2:8872150	unknown	none	TAAG>T	A1=76/R=7644	A1=1/R=3449	A1=77/R=11093	0.9845/0.029/0.6893	A1A1=5/A1R=66/RR=3789	A1A1=0/A1R=1/RR=1724	A1A1=5/A1R=67/RR=5513	17	KIDINS220	NM_020738.2	intron	NA	c.4054-41_4054-39del3	5316	0.235	0.88	unknown	unknown	TAAG	unknown	unknown	PASS	no	unknown	NA	NA	2:8732020
2:8873526	rs143441349	dbSNP_134	C>T	T=0/C=8202	T=26/C=3782	T=26/C=11984	0.0/0.6828/0.2165	TT=0/TC=0/CC=4101	TT=0/TC=26/CC=1878	TT=0/TC=26/CC=5979	50	KIDINS220	NM_020738.2	intron	NA	c.4053+48G>A	5316	0	-6.63	NA	unknown	C	C	unknown	PASS	no	unknown	NA	NA	2:8733396
2:8873550	rs202235222	dbSNP_137	T>C	C=7/T=8203	C=1/T=3785	C=8/T=11988	0.0853/0.0264/0.0667	CC=0/CT=7/TT=4098	CC=0/CT=1/TT=1892	CC=0/CT=8/TT=5990	68	KIDINS220	NM_020738.2	intron	NA	c.4053+24A>G	5316	0	0.88	NA	unknown	T	T	unknown	PASS	no	unknown	NA	NA	2:8733420
2:8873622	rs376711166	dbSNP_138	C>G	G=1/C=8315	G=0/C=3954	G=1/C=12269	0.012/0.0/0.0081	GG=0/GC=1/CC=4157	GG=0/GC=0/CC=1977	GG=0/GC=1/CC=6134	100	KIDINS220	NM_020738.2	coding-synonymous	p.(T1335=)	c.4005G>C	5316	0.04	-10.2	NA	unknown	C	C	unknown	PASS	no	unknown	NA	NA	2:8733492
2:8873623	rs146753792	dbSNP_134	G>A	A=0/G=8316	A=2/G=3956	A=2/G=12272	0.0/0.0505/0.0163	AA=0/AG=0/GG=4158	AA=0/AG=2/GG=1977	AA=0/AG=2/GG=6135	100	KIDINS220	NM_020738.2	missense	p.(T1335M)	c.4004C>T	5316	0.435	5.1	81	probably-damaging:0.968	G	G	unknown	PASS	no	unknown	NA	NA	2:8733493
2:8873630	rs373410380	dbSNP_138	G>A	A=1/G=8331	A=0/G=3966	A=1/G=12297	0.012/0.0/0.0081	AA=0/AG=1/GG=4165	AA=0/AG=0/GG=1983	AA=0/AG=1/GG=6148	100	KIDINS220	NM_020738.2	coding-synonymous	p.(L1333=)	c.3997C>T	5316	0.946	3.27	NA	unknown	G	G	unknown	PASS	no	unknown	NA	NA	2:8733500
2:8873652	rs377757996	dbSNP_138	T>C	C=1/T=8341	C=0/T=3998	C=1/T=12339	0.012/0.0/0.0081	CC=0/CT=1/TT=4170	CC=0/CT=0/TT=1999	CC=0/CT=1/TT=6169	94	KIDINS220	NM_020738.2	coding-synonymous	p.(T1325=)	c.3975A>G	5316	0.032	-10.2	NA	unknown	T	T	unknown	PASS	no	unknown	NA	NA	2:8733522
2:8873661	rs371285385	dbSNP_138	C>A	A=1/C=8353	A=0/C=4016	A=1/C=12369	0.012/0.0/0.0081	AA=0/AC=1/CC=4176	AA=0/AC=0/CC=2008	AA=0/AC=1/CC=6184	93	KIDINS220	NM_020738.2	coding-synonymous	p.(T1322=)	c.3966G>T	5316	0.001	-11	NA	unknown	C	C	unknown	PASS	no	unknown	NA	NA	2:8733531
2:8873678	rs374748463	dbSNP_138	C>T	T=1/C=8363	T=0/C=4044	T=1/C=12407	0.012/0.0/0.0081	TT=0/TC=1/CC=4181	TT=0/TC=0/CC=2022	TT=0/TC=1/CC=6203	86	KIDINS220	NM_020738.2	missense	p.(E1317K)	c.3949G>A	5316	0.952	5.49	56	probably-damaging:0.966	C	C	unknown	PASS	no	unknown	NA	NA	2:8733548
2:8873695	rs367743805	dbSNP_138	T>G	G=1/T=8385	G=0/T=4052	G=1/T=12437	0.0119/0.0/0.008	GG=0/GT=1/TT=4192	GG=0/GT=0/TT=2026	GG=0/GT=1/TT=6218	79	KIDINS220	NM_020738.2	missense	p.(N1311T)	c.3932A>C	5316	0	-10.6	65	benign:0.0	T	T	unknown	PASS	no	unknown	NA	NA	2:8733565
2:8873699	rs370461961	dbSNP_138	G>A	A=1/G=8391	A=0/G=4052	A=1/G=12443	0.0119/0.0/0.008	AA=0/AG=1/GG=4195	AA=0/AG=0/GG=2026	AA=0/AG=1/GG=6221	77	KIDINS220	NM_020738.2	missense	p.(H1310Y)	c.3928C>T	5316	0.329	5.49	83	possibly-damaging:0.579	G	G	unknown	PASS	no	unknown	NA	NA	2:8733569
2:8873706	rs76581861	dbSNP_131	G>A	A=1/G=8387	A=130/G=3952	A=131/G=12339	0.0119/3.1847/1.0505	AA=0/AG=1/GG=4193	AA=2/AG=126/GG=1913	AA=2/AG=127/GG=6106	73	KIDINS220	NM_020738.2	coding-synonymous	p.(R1307=)	c.3921C>T	5316	0	-3.78	NA	unknown	G	G	unknown	PASS	no	unknown	NA	NA	2:8733576
2:8873708	rs201958999	dbSNP_137	G>A	A=0/G=8390	A=7/G=4067	A=7/G=12457	0.0/0.1718/0.0562	AA=0/AG=0/GG=4195	AA=0/AG=7/GG=2030	AA=0/AG=7/GG=6225	71	KIDINS220	NM_020738.2	missense	p.(R1307C)	c.3919C>T	5316	0.003	4.6	180	benign:0.026	G	G	unknown	PASS	yes	unknown	NA	NA	2:8733578
2:8873728	rs372587282	dbSNP_138	G>A	A=0/G=8382	A=1/G=4057	A=1/G=12439	0.0/0.0246/0.008	AA=0/AG=0/GG=4191	AA=0/AG=1/GG=2028	AA=0/AG=1/GG=6219	60	KIDINS220	NM_020738.2	missense	p.(P1300L)	c.3899C>T	5316	0.009	5.18	98	possibly-damaging:0.628	G	G	unknown	PASS	no	unknown	NA	NA	2:8733598
2:8873731	rs76164009	dbSNP_132	G>C	C=0/G=8382	C=43/G=4029	C=43/G=12411	0.0/1.056/0.3453	CC=0/CG=0/GG=4191	CC=0/CG=43/GG=1993	CC=0/CG=43/GG=6184	59	KIDINS220	NM_020738.2	missense	p.(A1299G)	c.3896C>G	5316	0	-9.13	60	benign:0.142	G	G	unknown	PASS	yes	unknown	NA	NA	2:8733601
2:8873741	rs201077051	dbSNP_137	T>C	C=0/T=8388	C=3/T=4075	C=3/T=12463	0.0/0.0736/0.0241	CC=0/CT=0/TT=4194	CC=0/CT=3/TT=2036	CC=0/CT=3/TT=6230	58	KIDINS220	NM_020738.2	missense	p.(S1296G)	c.3886A>G	5316	0.001	-1.96	56	benign:0.123	T	T	unknown	PASS	yes	unknown	NA	NA	2:8733611
2:8873763	rs372288036	dbSNP_138	T>C	C=0/T=8368	C=1/T=4051	C=1/T=12419	0.0/0.0247/0.0081	CC=0/CT=0/TT=4184	CC=0/CT=1/TT=2025	CC=0/CT=1/TT=6209	54	KIDINS220	NM_020738.2	coding-synonymous	p.(P1288=)	c.3864A>G	5316	0.056	-10.9	NA	unknown	T	T	unknown	PASS	no	unknown	NA	NA	2:8733633
2:8873780	rs140417878	dbSNP_134	C>T	T=0/C=8344	T=5/C=3991	T=5/C=12335	0.0/0.1251/0.0405	TT=0/TC=0/CC=4172	TT=0/TC=5/CC=1993	TT=0/TC=5/CC=6165	50	KIDINS220	NM_020738.2	missense	p.(V1283M)	c.3847G>A	5316	0.813	4.73	21	possibly-damaging:0.582	C	C	unknown	PASS	yes	unknown	NA	NA	2:8733650
2:8873792	rs188547191	dbSNP_135	C>T	T=1/C=8297	T=1/C=3933	T=2/C=12230	0.0121/0.0254/0.0164	TT=0/TC=1/CC=4148	TT=0/TC=1/CC=1966	TT=0/TC=2/CC=6114	45	KIDINS220	NM_020738.2	missense	p.(A1279T)	c.3835G>A	5316	0.165	2.85	58	benign:0.005	C	C	unknown	PASS	yes	unknown	NA	NA	2:8733662
2:8873793	rs150396643	dbSNP_134	G>A	A=0/G=8290	A=21/G=3911	A=21/G=12201	0.0/0.5341/0.1718	AA=0/AG=0/GG=4145	AA=0/AG=21/GG=1945	AA=0/AG=21/GG=6090	44	KIDINS220	NM_020738.2	coding-synonymous	p.(N1278=)	c.3834C>T	5316	0.159	-0.02	NA	unknown	G	G	unknown	PASS	no	unknown	NA	NA	2:8733663
2:8873808	rs376288208	dbSNP_138	T>C	C=1/T=8261	C=0/T=3880	C=1/T=12141	0.0121/0.0/0.0082	CC=0/CT=1/TT=4130	CC=0/CT=0/TT=1940	CC=0/CT=1/TT=6070	39	KIDINS220	NM_020738.2	coding-synonymous	p.(V1273=)	c.3819A>G	5316	0.009	-4.43	NA	unknown	T	T	unknown	PASS	no	unknown	NA	NA	2:8733678
2:8873845	rs138168281	dbSNP_134	C>T	T=77/C=8077	T=11/C=3663	T=88/C=11740	0.9443/0.2994/0.744	TT=2/TC=73/CC=4002	TT=0/TC=11/CC=1826	TT=2/TC=84/CC=5828	20	KIDINS220	NM_020738.2	intron	NA	c.3817-35G>A	5316	0.001	1.84	NA	unknown	C	C	unknown	PASS	no	unknown	NA	NA	2:8733715
2:8873850	rs374005293	dbSNP_138	C>A	A=0/C=8138	A=1/C=3659	A=1/C=11797	0.0/0.0273/0.0085	AA=0/AC=0/CC=4069	AA=0/AC=1/CC=1829	AA=0/AC=1/CC=5898	18	KIDINS220	NM_020738.2	intron	NA	c.3817-40G>T	5316	0	0.31	NA	unknown	C	C	unknown	PASS	no	unknown	NA	NA	2:8733720
2:8874847	rs377648666	dbSNP_138	T>C	C=1/T=8181	C=0/T=3696	C=1/T=11877	0.0122/0.0/0.0084	CC=0/CT=1/TT=4090	CC=0/CT=0/TT=1848	CC=0/CT=1/TT=5938	98	KIDINS220	NM_020738.2	missense	p.(I1252V)	c.3754A>G	5316	1	5.96	29	benign:0.01	T	T	unknown	PASS	no	unknown	NA	NA	2:8734717
2:8874911	rs143217814	dbSNP_134	T>A	A=2/T=8134	A=45/T=3579	A=47/T=11713	0.0246/1.2417/0.3997	AA=0/AT=2/TT=4066	AA=0/AT=45/TT=1767	AA=0/AT=47/TT=5833	47	KIDINS220	NM_020738.2	intron	NA	c.3718-28A>T	5316	0	-10.7	NA	unknown	T	T	unknown	PASS	no	unknown	NA	NA	2:8734781
2:8876953	rs374606352	dbSNP_138	C>T	T=1/C=8343	T=0/C=4002	T=1/C=12345	0.012/0.0/0.0081	TT=0/TC=1/CC=4171	TT=0/TC=0/CC=2001	TT=0/TC=1/CC=6172	65	KIDINS220	NM_020738.2	intron	NA	c.3717+45G>A	5316	0	-3.2	NA	unknown	C	C	unknown	PASS	no	unknown	NA	NA	2:8736823
2:8876956	rs376102040	dbSNP_138	C>A	A=1/C=8343	A=0/C=4008	A=1/C=12351	0.012/0.0/0.0081	AA=0/AC=1/CC=4171	AA=0/AC=0/CC=2004	AA=0/AC=1/CC=6175	69	KIDINS220	NM_020738.2	intron	NA	c.3717+42G>T	5316	0	-7.83	NA	unknown	C	C	unknown	PASS	no	unknown	NA	NA	2:8736826
2:8876976	rs370563205	dbSNP_138	C>T	T=0/C=8346	T=2/C=4036	T=2/C=12382	0.0/0.0495/0.0161	TT=0/TC=0/CC=4173	TT=0/TC=2/CC=2017	TT=0/TC=2/CC=6190	80	KIDINS220	NM_020738.2	intron	NA	c.3717+22G>A	5316	0	-1.13	NA	unknown	C	C	unknown	PASS	no	unknown	NA	NA	2:8736846
2:8876979	rs373964493	dbSNP_138	G>A	A=1/G=8341	A=0/G=4030	A=1/G=12371	0.012/0.0/0.0081	AA=0/AG=1/GG=4170	AA=0/AG=0/GG=2015	AA=0/AG=1/GG=6185	84	KIDINS220	NM_020738.2	intron	NA	c.3717+19C>T	5316	0	-6.21	NA	unknown	G	G	unknown	PASS	no	unknown	NA	NA	2:8736849
2:8877036	rs367886108	dbSNP_138	G>C	C=3/G=8321	C=0/G=3994	C=3/G=12315	0.036/0.0/0.0244	CC=0/CG=3/GG=4159	CC=0/CG=0/GG=1997	CC=0/CG=3/GG=6156	116	KIDINS220	NM_020738.2	missense	p.(Q1227E)	c.3679C>G	5316	1	5.74	29	benign:0.214	G	G	unknown	PASS	no	unknown	NA	NA	2:8736906
2:8877042	rs61742863	dbSNP_129	G>A	A=651/G=7661	A=132/G=3832	A=783/G=11493	7.8321/3.33/6.3783	AA=26/AG=599/GG=3531	AA=2/AG=128/GG=1852	AA=28/AG=727/GG=5383	114	KIDINS220	NM_020738.2	coding-synonymous	p.(L1225=)	c.3673C>T	5316	1	3.91	NA	unknown	G	G	unknown	PASS	no	http://www.ncbi.nlm.nih.gov/pubmed?term=26148204	NA	NA	2:8736912
2:8877106	rs374208588	dbSNP_138	T>C	C=1/T=8245	C=0/T=3820	C=1/T=12065	0.0121/0.0/0.0083	CC=0/CT=1/TT=4122	CC=0/CT=0/TT=1910	CC=0/CT=1/TT=6032	75	KIDINS220	NM_020738.2	coding-synonymous	p.(P1203=)	c.3609A>G	5316	0	-3.8	NA	unknown	T	T	unknown	PASS	no	unknown	NA	NA	2:8736976
2:8877129	rs368373520	dbSNP_138	C>A	A=1/C=8235	A=0/C=3784	A=1/C=12019	0.0121/0.0/0.0083	AA=0/AC=1/CC=4117	AA=0/AC=0/CC=1892	AA=0/AC=1/CC=6009	60	KIDINS220	NM_020738.2	missense-near-splice	p.(G1196W)	c.3586G>T	5316	0.989	5.65	184	probably-damaging:0.98	C	C	unknown	PASS	no	unknown	NA	NA	2:8736999
2:8877136	rs139887022	dbSNP_134	A>G	G=30/A=8198	G=0/A=3768	G=30/A=11966	0.3646/0.0/0.2501	GG=0/GA=30/AA=4084	GG=0/GA=0/AA=1884	GG=0/GA=30/AA=5968	54	KIDINS220	NM_020738.2	intron	NA	c.3586-7T>C	5316	0	1.75	NA	unknown	A	A	unknown	PASS	no	unknown	NA	NA	2:8737006
2:8877159	rs375498327	dbSNP_138	G>A	A=0/G=8214	A=1/G=3747	A=1/G=11961	0.0/0.0267/0.0084	AA=0/AG=0/GG=4107	AA=0/AG=1/GG=1873	AA=0/AG=1/GG=5980	41	KIDINS220	NM_020738.2	intron	NA	c.3586-30C>T	5316	0.003	0.77	NA	unknown	G	G	unknown	PASS	no	unknown	NA	NA	2:8737029
2:8877161	rs367695837	dbSNP_138	A>C	C=1/A=8213	C=0/A=3746	C=1/A=11959	0.0122/0.0/0.0084	CC=0/CA=1/AA=4106	CC=0/CA=0/AA=1873	CC=0/CA=1/AA=5979	40	KIDINS220	NM_020738.2	intron	NA	c.3586-32T>G	5316	0	-0.54	NA	unknown	A	A	unknown	PASS	no	unknown	NA	NA	2:8737031
2:8887254	rs371912232	dbSNP_138	A>C	C=0/A=8398	C=1/A=4099	C=1/A=12497	0.0/0.0244/0.008	CC=0/CA=0/AA=4199	CC=0/CA=1/AA=2049	CC=0/CA=1/AA=6248	178	KIDINS220	NM_020738.2	intron	NA	c.3585+21T>G	5316	0.999	2.79	NA	unknown	A	A	unknown	PASS	no	unknown	NA	NA	2:8747124
2:8887313	rs374967193	dbSNP_138	C>G	G=1/C=8325	G=0/C=3940	G=1/C=12265	0.012/0.0/0.0082	GG=0/GC=1/CC=4162	GG=0/GC=0/CC=1970	GG=0/GC=1/CC=6132	168	KIDINS220	NM_020738.2	missense	p.(A1183P)	c.3547G>C	5316	1	5.12	27	possibly-damaging:0.855	C	C	unknown	PASS	no	unknown	NA	NA	2:8747183
2:8887343	rs369315481	dbSNP_138	A>G	G=0/A=8272	G=1/A=3825	G=1/A=12097	0.0/0.0261/0.0083	GG=0/GA=0/AA=4136	GG=0/GA=1/AA=1912	GG=0/GA=1/AA=6048	112	KIDINS220	NM_020738.2	intron	NA	c.3529-12T>C	5316	0.998	-0.55	NA	unknown	A	A	unknown	PASS	no	unknown	NA	NA	2:8747213
2:8887365	rs372112036	dbSNP_138	A>C	C=0/A=8250	C=2/A=3794	C=2/A=12044	0.0/0.0527/0.0166	CC=0/CA=0/AA=4125	CC=0/CA=2/AA=1896	CC=0/CA=2/AA=6021	81	KIDINS220	NM_020738.2	intron	NA	c.3529-34T>G	5316	0.001	2.34	NA	unknown	A	A	unknown	PASS	no	unknown	NA	NA	2:8747235
2:8887365	~rs34451528	dbSNP_126	AG>AGG;AG>A	A1=46/A2=4/R=7840	A1=36/A2=0/R=3608	A1=82/A2=4/R=11448	0.6337/0.9879/0.7456	A1A1=0/A1A2=0/A1R=46/A2A2=0/A2R=4/RR=3895	A1A1=3/A1A2=0/A1R=30/A2A2=0/A2R=0/RR=1789	A1A1=3/A1A2=0/A1R=76/A2A2=0/A2R=4/RR=5684	81	KIDINS220	NM_020738.2	intron	NA	c.3529-35_3529-34insC	5316	0.001	2.34	unknown	unknown	AG	unknown	unknown	PASS	no	unknown	NA	NA	2:8747235
2:8887365	~rs34451528	dbSNP_126	AG>AGG;AG>A	A1=46/A2=4/R=7840	A1=36/A2=0/R=3608	A1=82/A2=4/R=11448	0.6337/0.9879/0.7456	A1A1=0/A1A2=0/A1R=46/A2A2=0/A2R=4/RR=3895	A1A1=3/A1A2=0/A1R=30/A2A2=0/A2R=0/RR=1789	A1A1=3/A1A2=0/A1R=76/A2A2=0/A2R=4/RR=5684	81	KIDINS220	NM_020738.2	intron	NA	c.3529-35del1	5316	0.001	2.34	unknown	unknown	AG	unknown	unknown	PASS	no	unknown	NA	NA	2:8747235
2:8887371	unknown	none	A>AG	A1=4/R=7874	A1=0/R=3638	A1=4/R=11512	0.0508/0.0/0.0347	A1A1=0/A1R=4/RR=3935	A1A1=0/A1R=0/RR=1819	A1A1=0/A1R=4/RR=5754	76	KIDINS220	NM_020738.2	intron	NA	c.3529-41_3529-40insC	5316	0	-3.2	unknown	unknown	A	unknown	unknown	PASS	no	unknown	NA	NA	2:8747241
2:8887373	rs73912896	dbSNP_130	G>C	C=3/G=8243	C=100/G=3684	C=103/G=11927	0.0364/2.6427/0.8562	CC=0/CG=3/GG=4120	CC=0/CG=100/GG=1792	CC=0/CG=103/GG=5912	73	KIDINS220	NM_020738.2	intron	NA	c.3529-42C>G	5316	0	1.58	NA	unknown	G	G	unknown	PASS	no	unknown	NA	NA	2:8747243
2:8887374	rs369677862	dbSNP_138	G>C	C=1/G=8245	C=0/G=3782	C=1/G=12027	0.0121/0.0/0.0083	CC=0/CG=1/GG=4122	CC=0/CG=0/GG=1891	CC=0/CG=1/GG=6013	72	KIDINS220	NM_020738.2	intron	NA	c.3529-43C>G	5316	0	-4.5	NA	unknown	G	G	unknown	PASS	no	unknown	NA	NA	2:8747244
2:8887404	rs60592986	dbSNP_129	T>C	C=2/T=3180	C=165/T=1219	C=167/T=4399	0.0629/11.922/3.6575	CC=0/CT=2/TT=1589	CC=11/CT=143/TT=538	CC=11/CT=145/TT=2127	31	KIDINS220	NM_020738.2	intron	NA	c.3529-73A>G	5316	0	-4.07	NA	unknown	T	T	unknown	PASS	no	unknown	NA	NA	2:8747274
2:8887965	rs73912897	dbSNP_130	T>C	C=1/T=3981	C=27/T=1717	C=28/T=5698	0.0251/1.5482/0.489	CC=0/CT=1/TT=1990	CC=0/CT=27/TT=845	CC=0/CT=28/TT=2835	20	KIDINS220	NM_020738.2	intron	NA	c.3528+52A>G	5316	0.011	4.35	NA	unknown	T	T	unknown	PASS	no	unknown	NA	NA	2:8747835
2:8887981	unknown	none	TA>T	A1=10/R=7804	A1=4/R=3482	A1=14/R=11286	0.128/0.1147/0.1239	A1A1=4/A1R=2/RR=3901	A1A1=2/A1R=0/RR=1741	A1A1=6/A1R=2/RR=5642	39	KIDINS220	NM_020738.2	intron	NA	c.3528+35del1	5316	0.657	4.05	unknown	unknown	TA	unknown	unknown	PASS	no	unknown	NA	NA	2:8747851
2:8887995	rs369934961	dbSNP_138	C>T	T=1/C=8149	T=0/C=3610	T=1/C=11759	0.0123/0.0/0.0085	TT=0/TC=1/CC=4074	TT=0/TC=0/CC=1805	TT=0/TC=1/CC=5879	56	KIDINS220	NM_020738.2	intron	NA	c.3528+22G>A	5316	0.036	-1.92	NA	unknown	C	C	unknown	PASS	no	unknown	NA	NA	2:8747865
2:8887996	rs200625902	dbSNP_137	G>A	A=0/G=8150	A=1/G=3613	A=1/G=11763	0.0/0.0277/0.0085	AA=0/AG=0/GG=4075	AA=0/AG=1/GG=1806	AA=0/AG=1/GG=5881	61	KIDINS220	NM_020738.2	intron	NA	c.3528+21C>T	5316	0.176	0.05	NA	unknown	G	G	unknown	PASS	no	unknown	NA	NA	2:8747866
2:8888035	rs376251165	dbSNP_138	T>C	C=1/T=8171	C=0/T=3652	C=1/T=11823	0.0122/0.0/0.0085	CC=0/CT=1/TT=4085	CC=0/CT=0/TT=1826	CC=0/CT=1/TT=5911	121	KIDINS220	NM_020738.2	coding-synonymous	p.(R1170=)	c.3510A>G	5316	1	4.59	NA	unknown	T	T	unknown	PASS	no	unknown	NA	NA	2:8747905
2:8888064	rs370341892	dbSNP_138	G>A	A=1/G=8183	A=0/G=3662	A=1/G=11845	0.0122/0.0/0.0084	AA=0/AG=1/GG=4091	AA=0/AG=0/GG=1831	AA=0/AG=1/GG=5922	126	KIDINS220	NM_020738.2	missense	p.(R1161C)	c.3481C>T	5316	1	5.75	180	probably-damaging:1.0	G	G	unknown	PASS	no	unknown	NA	NA	2:8747934
2:8888085	rs374088731	dbSNP_138	C>T	T=1/C=8185	T=0/C=3658	T=1/C=11843	0.0122/0.0/0.0084	TT=0/TC=1/CC=4092	TT=0/TC=0/CC=1829	TT=0/TC=1/CC=5921	119	KIDINS220	NM_020738.2	missense	p.(G1154S)	c.3460G>A	5316	0.021	-3.31	56	benign:0.001	C	C	unknown	PASS	no	unknown	NA	NA	2:8747955
2:8888086	rs199913532	dbSNP_137	G>A	A=23/G=8161	A=2/G=3658	A=25/G=11819	0.281/0.0546/0.2111	AA=0/AG=23/GG=4069	AA=0/AG=2/GG=1828	AA=0/AG=25/GG=5897	118	KIDINS220	NM_020738.2	coding-synonymous	p.(G1153=)	c.3459C>T	5316	0.972	5.75	NA	unknown	G	G	unknown	PASS	no	http://www.ncbi.nlm.nih.gov/pubmed?term=28552196	NA	NA	2:8747956
2:8888106	rs374514933	dbSNP_138	T>C	C=0/T=8168	C=1/T=3645	C=1/T=11813	0.0/0.0274/0.0085	CC=0/CT=0/TT=4084	CC=0/CT=1/TT=1822	CC=0/CT=1/TT=5906	114	KIDINS220	NM_020738.2	missense	p.(T1147A)	c.3439A>G	5316	1	3.15	58	benign:0.047	T	T	unknown	PASS	no	unknown	NA	NA	2:8747976
2:8888107	rs368708454	dbSNP_138	G>A	A=1/G=8167	A=0/G=3642	A=1/G=11809	0.0122/0.0/0.0085	AA=0/AG=1/GG=4083	AA=0/AG=0/GG=1821	AA=0/AG=1/GG=5904	113	KIDINS220	NM_020738.2	coding-synonymous	p.(Y1146=)	c.3438C>T	5316	1	3.96	NA	unknown	G	G	unknown	PASS	no	unknown	NA	NA	2:8747977
2:8888112	rs372306517	dbSNP_138	G>T	T=1/G=8167	T=0/G=3640	T=1/G=11807	0.0122/0.0/0.0085	TT=0/TG=1/GG=4083	TT=0/TG=0/GG=1820	TT=0/TG=1/GG=5903	113	KIDINS220	NM_020738.2	missense	p.(L1145I)	c.3433C>A	5316	1	3.85	5	benign:0.008	G	G	unknown	PASS	no	unknown	NA	NA	2:8747982
2:8888118	rs374204018	dbSNP_138	G>A	A=1/G=8167	A=0/G=3650	A=1/G=11817	0.0122/0.0/0.0085	AA=0/AG=1/GG=4083	AA=0/AG=0/GG=1825	AA=0/AG=1/GG=5908	109	KIDINS220	NM_020738.2	missense	p.(P1143S)	c.3427C>T	5316	1	5.75	74	probably-damaging:1.0	G	G	unknown	PASS	no	unknown	NA	NA	2:8747988
2:8888149	unknown	none	CA>C	A1=0/R=7820	A1=2/R=3522	A1=2/R=11342	0.0/0.0568/0.0176	A1A1=0/A1R=0/RR=3910	A1A1=0/A1R=2/RR=1760	A1A1=0/A1R=2/RR=5670	74	KIDINS220	NM_020738.2	intron	NA	c.3415-20del1	5316	1	5.76	unknown	unknown	CA	unknown	unknown	PASS	no	unknown	NA	NA	2:8748019
2:8888167	rs367845055	dbSNP_138	T>G	G=0/T=8146	G=1/T=3643	G=1/T=11789	0.0/0.0274/0.0085	GG=0/GT=0/TT=4073	GG=0/GT=1/TT=1821	GG=0/GT=1/TT=5894	57	KIDINS220	NM_020738.2	intron	NA	c.3415-37A>C	5316	1	5.76	NA	unknown	T	T	unknown	PASS	no	unknown	NA	NA	2:8748037
2:8890265	rs371987705	dbSNP_138	G>A	A=0/G=8330	A=1/G=3941	A=1/G=12271	0.0/0.0254/0.0081	AA=0/AG=0/GG=4165	AA=0/AG=1/GG=1970	AA=0/AG=1/GG=6135	56	KIDINS220	NM_020738.2	missense	p.(P1131S)	c.3391C>T	5316	0.705	5.79	74	probably-damaging:1.0	G	G	unknown	PASS	no	unknown	NA	NA	2:8750135
2:8890269	rs376957194	dbSNP_138	C>T	T=0/C=8336	T=1/C=3969	T=1/C=12305	0.0/0.0252/0.0081	TT=0/TC=0/CC=4168	TT=0/TC=1/CC=1984	TT=0/TC=1/CC=6152	58	KIDINS220	NM_020738.2	coding-synonymous	p.(T1129=)	c.3387G>A	5316	0.655	-5.4	NA	unknown	C	C	unknown	PASS	no	unknown	NA	NA	2:8750139
2:8890312	rs369550617	dbSNP_138	A>C	C=1/A=8405	C=0/A=4072	C=1/A=12477	0.0119/0.0/0.008	CC=0/CA=1/AA=4202	CC=0/CA=0/AA=2036	CC=0/CA=1/AA=6238	72	KIDINS220	NM_020738.2	missense	p.(V1115G)	c.3344T>G	5316	0.948	5.79	109	possibly-damaging:0.93	A	A	unknown	PASS	no	unknown	NA	NA	2:8750182
2:8890314	rs372263522	dbSNP_138	T>C	C=1/T=8405	C=0/T=4070	C=1/T=12475	0.0119/0.0/0.008	CC=0/CT=1/TT=4202	CC=0/CT=0/TT=2035	CC=0/CT=1/TT=6237	72	KIDINS220	NM_020738.2	coding-synonymous	p.(G1114=)	c.3342A>G	5316	0.864	-4.18	NA	unknown	T	T	unknown	PASS	no	unknown	NA	NA	2:8750184
2:8890323	rs375561088	dbSNP_138	G>A	A=1/G=8385	A=0/G=4054	A=1/G=12439	0.0119/0.0/0.008	AA=0/AG=1/GG=4192	AA=0/AG=0/GG=2027	AA=0/AG=1/GG=6219	74	KIDINS220	NM_020738.2	coding-synonymous	p.(F1111=)	c.3333C>T	5316	0	-5.66	NA	unknown	G	G	unknown	PASS	no	unknown	NA	NA	2:8750193
2:8890333	rs145408226	dbSNP_134	T>C	C=20/T=8352	C=2/T=4054	C=22/T=12406	0.2389/0.0493/0.177	CC=0/CT=20/TT=4166	CC=0/CT=2/TT=2026	CC=0/CT=22/TT=6192	77	KIDINS220	NM_020738.2	missense	p.(N1108S)	c.3323A>G	5316	0.741	-0.04	46	benign:0.0	T	T	unknown	PASS	yes	unknown	NA	NA	2:8750203
2:8890377	rs373472346	dbSNP_138	T>A	A=1/T=8315	A=0/T=3980	A=1/T=12295	0.012/0.0/0.0081	AA=0/AT=1/TT=4157	AA=0/AT=0/TT=1990	AA=0/AT=1/TT=6147	71	KIDINS220	NM_020738.2	coding-synonymous	p.(S1093=)	c.3279A>T	5316	0	-11.6	NA	unknown	T	T	unknown	PASS	no	unknown	NA	NA	2:8750247
2:8890385	rs199962701	dbSNP_137	C>A	A=2/C=8288	A=1/C=3969	A=3/C=12257	0.0241/0.0252/0.0245	AA=0/AC=2/CC=4143	AA=0/AC=1/CC=1984	AA=0/AC=3/CC=6127	66	KIDINS220	NM_020738.2	missense	p.(A1091S)	c.3271G>T	5316	0	0.26	99	benign:0.015	C	C	unknown	PASS	yes	unknown	NA	NA	2:8750255
2:8890399	rs369018621	dbSNP_138	T>C	C=0/T=8266	C=1/T=3939	C=1/T=12205	0.0/0.0254/0.0082	CC=0/CT=0/TT=4133	CC=0/CT=1/TT=1969	CC=0/CT=1/TT=6102	58	KIDINS220	NM_020738.2	missense	p.(E1086G)	c.3257A>G	5316	0.189	4.59	98	possibly-damaging:0.507	T	T	unknown	PASS	no	unknown	NA	NA	2:8750269
2:8890408	rs372824408	dbSNP_138	G>C	C=1/G=8259	C=0/G=3916	C=1/G=12175	0.0121/0.0/0.0082	CC=0/CG=1/GG=4129	CC=0/CG=0/GG=1958	CC=0/CG=1/GG=6087	53	KIDINS220	NM_020738.2	missense	p.(P1083R)	c.3248C>G	5316	0.221	4.88	103	possibly-damaging:0.919	G	G	unknown	PASS	no	unknown	NA	NA	2:8750278
2:8890413	rs376079231	dbSNP_138	C>T	T=1/C=8263	T=0/C=3920	T=1/C=12183	0.0121/0.0/0.0082	TT=0/TC=1/CC=4131	TT=0/TC=0/CC=1960	TT=0/TC=1/CC=6091	49	KIDINS220	NM_020738.2	coding-synonymous	p.(P1081=)	c.3243G>A	5316	0.015	-8.64	NA	unknown	C	C	unknown	PASS	no	unknown	NA	NA	2:8750283
2:8890422	rs370476082	dbSNP_138	C>T	T=0/C=8272	T=1/C=3917	T=1/C=12189	0.0/0.0255/0.0082	TT=0/TC=0/CC=4136	TT=0/TC=1/CC=1958	TT=0/TC=1/CC=6094	44	KIDINS220	NM_020738.2	coding-synonymous	p.(A1078=)	c.3234G>A	5316	0	-11.6	NA	unknown	C	C	unknown	PASS	no	unknown	NA	NA	2:8750292
2:8890428	rs375000969	dbSNP_138	T>C	C=0/T=8268	C=2/T=3898	C=2/T=12166	0.0/0.0513/0.0164	CC=0/CT=0/TT=4134	CC=0/CT=2/TT=1948	CC=0/CT=2/TT=6082	39	KIDINS220	NM_020738.2	coding-synonymous	p.(G1076=)	c.3228A>G	5316	0.004	-11.6	NA	unknown	T	T	unknown	PASS	no	unknown	NA	NA	2:8750298
2:8890440	rs376785899	dbSNP_138	G>T	T=1/G=8279	T=0/G=3898	T=1/G=12177	0.0121/0.0/0.0082	TT=0/TG=1/GG=4139	TT=0/TG=0/GG=1949	TT=0/TG=1/GG=6088	34	KIDINS220	NM_020738.2	coding-synonymous	p.(I1072=)	c.3216C>A	5316	0.594	-1.93	NA	unknown	G	G	unknown	PASS	no	unknown	NA	NA	2:8750310
2:8890505	rs76429367	dbSNP_132	A>G	G=0/A=8252	G=73/A=3753	G=73/A=12005	0.0/1.908/0.6044	GG=0/GA=0/AA=4126	GG=1/GA=71/AA=1841	GG=1/GA=71/AA=5967	13	KIDINS220	NM_020738.2	intron	NA	c.3191-40T>C	5316	0.001	2.58	NA	unknown	A	A	unknown	PASS	no	unknown	NA	NA	2:8750375
2:8891556	rs374689815	dbSNP_138	G>A	A=1/G=8115	A=0/G=3580	A=1/G=11695	0.0123/0.0/0.0085	AA=0/AG=1/GG=4057	AA=0/AG=0/GG=1790	AA=0/AG=1/GG=5847	32	KIDINS220	NM_020738.2	intron	NA	c.3190+40C>T	5316	0.004	2.19	NA	unknown	G	G	unknown	PASS	no	unknown	NA	NA	2:8751426
2:8891567	rs367683465	dbSNP_138	G>T	T=1/G=8111	T=0/G=3580	T=1/G=11691	0.0123/0.0/0.0086	TT=0/TG=1/GG=4055	TT=0/TG=0/GG=1790	TT=0/TG=1/GG=5845	39	KIDINS220	NM_020738.2	intron	NA	c.3190+29C>A	5316	0	-0.2	NA	unknown	G	G	unknown	PASS	no	unknown	NA	NA	2:8751437
2:8891611	rs184866499	dbSNP_135	G>A	A=1/G=8135	A=0/G=3610	A=1/G=11745	0.0123/0.0/0.0085	AA=0/AG=1/GG=4067	AA=0/AG=0/GG=1805	AA=0/AG=1/GG=5872	87	KIDINS220	NM_020738.2	missense	p.(R1059W)	c.3175C>T	5316	0.978	5.07	101	probably-damaging:1.0	G	G	unknown	PASS	yes	unknown	NA	NA	2:8751481
2:8891635	rs374898741	dbSNP_138	T>A	A=1/T=8135	A=0/T=3634	A=1/T=11769	0.0123/0.0/0.0085	AA=0/AT=1/TT=4067	AA=0/AT=0/TT=1817	AA=0/AT=1/TT=5884	99	KIDINS220	NM_020738.2	missense	p.(T1051S)	c.3151A>T	5316	0.995	5.07	58	probably-damaging:1.0	T	T	unknown	PASS	no	unknown	NA	NA	2:8751505
2:8891642	rs367860069	dbSNP_138	C>T	T=1/C=8141	T=0/C=3640	T=1/C=11781	0.0123/0.0/0.0085	TT=0/TC=1/CC=4070	TT=0/TC=0/CC=1820	TT=0/TC=1/CC=5890	98	KIDINS220	NM_020738.2	coding-synonymous	p.(L1048=)	c.3144G>A	5316	0.979	5.07	NA	unknown	C	C	unknown	PASS	no	unknown	NA	NA	2:8751512
2:8891714	rs372134421	dbSNP_138	A>G	G=11/A=8131	G=0/A=3618	G=11/A=11749	0.1351/0.0/0.0935	GG=0/GA=11/AA=4060	GG=0/GA=0/AA=1809	GG=0/GA=11/AA=5869	97	KIDINS220	NM_020738.2	coding-synonymous	p.(D1024=)	c.3072T>C	5316	0.996	-1.79	NA	unknown	A	A	unknown	PASS	no	unknown	NA	NA	2:8751584
2:8891764	rs376355404	dbSNP_138	T>G	G=1/T=8171	G=0/T=3642	G=1/T=11813	0.0122/0.0/0.0085	GG=0/GT=1/TT=4085	GG=0/GT=0/TT=1821	GG=0/GT=1/TT=5906	64	KIDINS220	NM_020738.2	missense	p.(N1008H)	c.3022A>C	5316	1	5.08	68	probably-damaging:1.0	T	T	unknown	PASS	no	unknown	NA	NA	2:8751634
2:8891775	unknown	none	C>CT	A1=1/R=7831	A1=3/R=3533	A1=4/R=11364	0.0128/0.0848/0.0352	A1A1=0/A1R=1/RR=3915	A1A1=0/A1R=3/RR=1765	A1A1=0/A1R=4/RR=5680	55	KIDINS220	NM_020738.2	splice-3	NA	c.3012-2_3012-1insA	5316	0.996	5.08	unknown	unknown	C	unknown	unknown	PASS	no	unknown	NA	NA	2:8751645
2:8891778	rs368459869	dbSNP_138	C>T	T=0/C=8164	T=2/C=3652	T=2/C=11816	0.0/0.0547/0.0169	TT=0/TC=0/CC=4082	TT=0/TC=2/CC=1825	TT=0/TC=2/CC=5907	53	KIDINS220	NM_020738.2	intron	NA	c.3012-4G>A	5316	0.352	0.36	NA	unknown	C	C	unknown	PASS	no	unknown	NA	NA	2:8751648
2:8910789	rs372710590	dbSNP_138	A>G	G=1/A=8151	G=0/A=3608	G=1/A=11759	0.0123/0.0/0.0085	GG=0/GA=1/AA=4075	GG=0/GA=0/AA=1804	GG=0/GA=1/AA=5879	57	KIDINS220	NM_020738.2	intron	NA	c.3011+11T>C	5316	0	0.64	NA	unknown	A	A	unknown	PASS	no	unknown	NA	NA	2:8770659
2:8910792	rs375999474	dbSNP_138	A>G	G=0/A=8154	G=1/A=3609	G=1/A=11763	0.0/0.0277/0.0085	GG=0/GA=0/AA=4077	GG=0/GA=1/AA=1804	GG=0/GA=1/AA=5881	60	KIDINS220	NM_020738.2	intron	NA	c.3011+8T>C	5316	0	-7.71	NA	unknown	A	A	unknown	PASS	no	unknown	NA	NA	2:8770662
2:8910812	rs368903999	dbSNP_138	G>A	A=0/G=8180	A=1/G=3653	A=1/G=11833	0.0/0.0274/0.0085	AA=0/AG=0/GG=4090	AA=0/AG=1/GG=1826	AA=0/AG=1/GG=5916	80	KIDINS220	NM_020738.2	missense	p.(T1000I)	c.2999C>T	5316	1	5.43	89	probably-damaging:0.996	G	G	unknown	PASS	no	unknown	NA	NA	2:8770682
2:8910890	rs201780168	dbSNP_137	T>A	A=2/T=8234	A=1/T=3775	A=3/T=12009	0.0243/0.0265/0.025	AA=0/AT=2/TT=4116	AA=0/AT=1/TT=1887	AA=0/AT=3/TT=6003	88	KIDINS220	NM_020738.2	missense	p.(Q974L)	c.2921A>T	5316	1	5.43	113	probably-damaging:0.991	T	T	unknown	PASS	yes	unknown	NA	NA	2:8770760
2:8910940	rs182521570	dbSNP_135	C>G	G=1/C=8195	G=0/C=3714	G=1/C=11909	0.0122/0.0/0.0084	GG=0/GC=1/CC=4097	GG=0/GC=0/CC=1857	GG=0/GC=1/CC=5954	86	KIDINS220	NM_020738.2	missense	p.(Q957H)	c.2871G>C	5316	1	3.49	24	benign:0.076	C	C	unknown	PASS	no	unknown	NA	NA	2:8770810
2:8910987	rs12476359	dbSNP_120	A>T	T=7973/A=157	T=3493/A=91	T=11466/A=248	1.9311/2.5391/2.1171	TT=3911/TA=151/AA=3	TT=1702/TA=89/AA=1	TT=5613/TA=240/AA=4	39	KIDINS220	NM_020738.2	intron	NA	c.2849-25T>A	5316	0	-9.87	NA	unknown	A	T	unknown	PASS	no	unknown	NA	NA	2:8770857
2:8911010	rs17630893	dbSNP_123	T>C	C=4731/T=3379	C=706/T=2866	C=5437/T=6245	41.6646/19.7648/46.5417	CC=1375/CT=1981/TT=699	CC=82/CT=542/TT=1162	CC=1457/CT=2523/TT=1861	29	KIDINS220	NM_020738.2	intron	NA	c.2849-48A>G	5316	0.001	-0.69	NA	unknown	T	T	unknown	PASS	no	unknown	NA	NA	2:8770880
2:8916847	rs4669338	dbSNP_111	A>T	T=8003/A=159	T=3150/A=512	T=11153/A=671	1.9481/13.9814/5.6749	TT=3923/TA=157/AA=1	TT=1360/TA=430/AA=41	TT=5283/TA=587/AA=42	44	KIDINS220	NM_020738.2	intron	NA	c.2848+31T>A	5316	0	1.51	NA	unknown	A	T	unknown	PASS	no	unknown	NA	NA	2:8776717
2:8916919	rs202054411	dbSNP_137	G>C	C=1/G=8251	C=0/G=3798	C=1/G=12049	0.0121/0.0/0.0083	CC=0/CG=1/GG=4125	CC=0/CG=0/GG=1899	CC=0/CG=1/GG=6024	96	KIDINS220	NM_020738.2	missense	p.(P936R)	c.2807C>G	5316	0.989	5.62	103	probably-damaging:0.999	G	G	unknown	PASS	yes	unknown	NA	NA	2:8776789
2:8916945	rs2304590	dbSNP_100	G>A	A=2/G=8292	A=0/G=3862	A=2/G=12154	0.0241/0.0/0.0165	AA=0/AG=2/GG=4145	AA=0/AG=0/GG=1931	AA=0/AG=2/GG=6076	107	KIDINS220	NM_020738.2	coding-synonymous	p.(T927=)	c.2781C>T	5316	0.375	-11.2	NA	unknown	G	G	unknown	PASS	no	unknown	NA	NA	2:8776815
2:8916983	rs377694962	dbSNP_138	G>A	A=0/G=8296	A=3/G=3905	A=3/G=12201	0.0/0.0768/0.0246	AA=0/AG=0/GG=4148	AA=0/AG=3/GG=1951	AA=0/AG=3/GG=6099	118	KIDINS220	NM_020738.2	missense	p.(R915C)	c.2743C>T	5316	0.855	4.72	180	probably-damaging:1.0	G	G	unknown	PASS	no	unknown	NA	NA	2:8776853
2:8917012	rs371277615	dbSNP_138	C>T	T=1/C=8291	T=0/C=3908	T=1/C=12199	0.0121/0.0/0.0082	TT=0/TC=1/CC=4145	TT=0/TC=0/CC=1954	TT=0/TC=1/CC=6099	122	KIDINS220	NM_020738.2	missense	p.(R905Q)	c.2714G>A	5316	0.99	5.62	43	probably-damaging:0.997	C	C	unknown	PASS	no	unknown	NA	NA	2:8776882
2:8917035	rs200258346	dbSNP_137	G>A	A=0/G=8284	A=2/G=3864	A=2/G=12148	0.0/0.0517/0.0165	AA=0/AG=0/GG=4142	AA=0/AG=2/GG=1931	AA=0/AG=2/GG=6073	111	KIDINS220	NM_020738.2	intron	NA	c.2704-13C>T	5316	0	-0.6	NA	unknown	G	G	unknown	PASS	no	unknown	NA	NA	2:8776905
2:8917037	rs367747789	dbSNP_138	C>T	T=1/C=8283	T=0/C=3860	T=1/C=12143	0.0121/0.0/0.0082	TT=0/TC=1/CC=4141	TT=0/TC=0/CC=1930	TT=0/TC=1/CC=6071	109	KIDINS220	NM_020738.2	intron	NA	c.2704-15G>A	5316	0	-3.95	NA	unknown	C	C	unknown	PASS	no	unknown	NA	NA	2:8776907
2:8917057	rs370698109	dbSNP_138	C>T	T=0/C=8252	T=1/C=3797	T=1/C=12049	0.0/0.0263/0.0083	TT=0/TC=0/CC=4126	TT=0/TC=1/CC=1898	TT=0/TC=1/CC=6024	91	KIDINS220	NM_020738.2	intron	NA	c.2704-35G>A	5316	0	-2.16	NA	unknown	C	C	unknown	PASS	no	unknown	NA	NA	2:8776927
2:8918718	rs374061373	dbSNP_138	T>C	C=0/T=8192	C=5/T=3701	C=5/T=11893	0.0/0.1349/0.042	CC=0/CT=0/TT=4096	CC=0/CT=5/TT=1848	CC=0/CT=5/TT=5944	69	KIDINS220	NM_020738.2	intron	NA	c.2703+51A>G	5316	0.006	0.95	NA	unknown	T	T	unknown	PASS	no	unknown	NA	NA	2:8778588
2:8918762	rs368450887	dbSNP_138	A>G	G=1/A=8223	G=0/A=3766	G=1/A=11989	0.0122/0.0/0.0083	GG=0/GA=1/AA=4111	GG=0/GA=0/AA=1883	GG=0/GA=1/AA=5994	143	KIDINS220	NM_020738.2	intron	NA	c.2703+7T>C	5316	0.005	-1.91	NA	unknown	A	A	unknown	PASS	no	unknown	NA	NA	2:8778632
2:8918771	rs372110208	dbSNP_138	G>A	A=1/G=8225	A=0/G=3772	A=1/G=11997	0.0122/0.0/0.0083	AA=0/AG=1/GG=4112	AA=0/AG=0/GG=1886	AA=0/AG=1/GG=5998	152	KIDINS220	NM_020738.2	missense	p.(R901W)	c.2701C>T	5316	0.992	4.96	101	probably-damaging:1.0	G	G	unknown	PASS	no	unknown	NA	NA	2:8778641
2:8918841	rs376503483	dbSNP_138	A>G	G=1/A=8223	G=0/A=3764	G=1/A=11987	0.0122/0.0/0.0083	GG=0/GA=1/AA=4111	GG=0/GA=0/AA=1882	GG=0/GA=1/AA=5993	154	KIDINS220	NM_020738.2	coding-synonymous	p.(A877=)	c.2631T>C	5316	0.616	2.14	NA	unknown	A	A	unknown	PASS	no	unknown	NA	NA	2:8778711
2:8919003	rs368481934	dbSNP_138	T>C	C=1/T=8239	C=0/T=3812	C=1/T=12051	0.0121/0.0/0.0083	CC=0/CT=1/TT=4119	CC=0/CT=0/TT=1906	CC=0/CT=1/TT=6025	112	KIDINS220	NM_020738.2	intron	NA	c.2614+23A>G	5316	0	-5.15	NA	unknown	T	T	unknown	PASS	no	unknown	NA	NA	2:8778873
2:8919008	rs372291689	dbSNP_138	T>C	C=1/T=8237	C=0/T=3814	C=1/T=12051	0.0121/0.0/0.0083	CC=0/CT=1/TT=4118	CC=0/CT=0/TT=1907	CC=0/CT=1/TT=6025	116	KIDINS220	NM_020738.2	intron	NA	c.2614+18A>G	5316	0	-5.77	NA	unknown	T	T	unknown	PASS	no	unknown	NA	NA	2:8778878
2:8919036	rs201853476	dbSNP_137	T>C	C=0/T=8270	C=1/T=3825	C=1/T=12095	0.0/0.0261/0.0083	CC=0/CT=0/TT=4135	CC=0/CT=1/TT=1912	CC=0/CT=1/TT=6047	141	KIDINS220	NM_020738.2	coding-synonymous	p.(S868=)	c.2604A>G	5316	0.005	-8.38	NA	unknown	T	T	unknown	PASS	no	unknown	NA	NA	2:8778906
2:8919047	rs369028313	dbSNP_138	C>T	T=0/C=8276	T=3/C=3831	T=3/C=12107	0.0/0.0782/0.0248	TT=0/TC=0/CC=4138	TT=0/TC=3/CC=1914	TT=0/TC=3/CC=6052	155	KIDINS220	NM_020738.2	missense	p.(V865I)	c.2593G>A	5316	0.961	-1.63	29	benign:0.003	C	C	unknown	PASS	no	unknown	NA	NA	2:8778917
2:8919048	rs200719766	dbSNP_137	G>A	A=4/G=8272	A=0/G=3828	A=4/G=12100	0.0483/0.0/0.033	AA=0/AG=4/GG=4134	AA=0/AG=0/GG=1914	AA=0/AG=4/GG=6048	158	KIDINS220	NM_020738.2	coding-synonymous	p.(D864=)	c.2592C>T	5316	0.974	1.21	NA	unknown	G	G	unknown	PASS	no	unknown	NA	NA	2:8778918
2:8919061	rs375382643	dbSNP_138	G>C	C=1/G=8281	C=0/G=3820	C=1/G=12101	0.0121/0.0/0.0083	CC=0/CG=1/GG=4140	CC=0/CG=0/GG=1910	CC=0/CG=1/GG=6050	162	KIDINS220	NM_020738.2	missense	p.(A860G)	c.2579C>G	5316	0.032	5.64	60	benign:0.165	G	G	unknown	PASS	no	unknown	NA	NA	2:8778931
2:8919071	rs200104720	dbSNP_137	C>T	T=7/C=8267	T=1/C=3825	T=8/C=12092	0.0846/0.0261/0.0661	TT=0/TC=7/CC=4130	TT=0/TC=1/CC=1912	TT=0/TC=8/CC=6042	168	KIDINS220	NM_020738.2	missense	p.(V857I)	c.2569G>A	5316	0.835	5.64	29	benign:0.014	C	C	unknown	PASS	yes	unknown	NA	NA	2:8778941
2:8919072	rs61742927	dbSNP_129	G>A	A=1/G=8271	A=78/G=3742	A=79/G=12013	0.0121/2.0419/0.6533	AA=0/AG=1/GG=4135	AA=0/AG=78/GG=1832	AA=0/AG=79/GG=5967	168	KIDINS220	NM_020738.2	coding-synonymous	p.(L856=)	c.2568C>T	5316	0.748	-3.86	NA	unknown	G	G	unknown	PASS	no	unknown	NA	NA	2:8778942
2:8919087	rs377667176	dbSNP_138	A>G	G=4/A=8274	G=0/A=3820	G=4/A=12094	0.0483/0.0/0.0331	GG=0/GA=4/AA=4135	GG=0/GA=0/AA=1910	GG=0/GA=4/AA=6045	185	KIDINS220	NM_020738.2	coding-synonymous	p.(N851=)	c.2553T>C	5316	0.698	-0.03	NA	unknown	A	A	unknown	PASS	no	unknown	NA	NA	2:8778957
2:8919106	rs371161832	dbSNP_138	T>C	C=1/T=8265	C=0/T=3806	C=1/T=12071	0.0121/0.0/0.0083	CC=0/CT=1/TT=4132	CC=0/CT=0/TT=1903	CC=0/CT=1/TT=6035	206	KIDINS220	NM_020738.2	missense	p.(N845S)	c.2534A>G	5316	0.319	5.64	46	probably-damaging:0.999	T	T	unknown	PASS	no	unknown	NA	NA	2:8778976
2:8919243	rs374611448	dbSNP_138	C>T	T=1/C=8179	T=0/C=3668	T=1/C=11847	0.0122/0.0/0.0084	TT=0/TC=1/CC=4089	TT=0/TC=0/CC=1834	TT=0/TC=1/CC=5923	119	KIDINS220	NM_020738.2	coding-synonymous	p.(P799=)	c.2397G>A	5316	0.492	-10.4	NA	unknown	C	C	unknown	PASS	no	unknown	NA	NA	2:8779113
2:8919276	rs114946239	dbSNP_132	C>T	T=0/C=8176	T=16/C=3640	T=16/C=11816	0.0/0.4376/0.1352	TT=0/TC=0/CC=4088	TT=0/TC=16/CC=1812	TT=0/TC=16/CC=5900	72	KIDINS220	NM_020738.2	intron	NA	c.2371-7G>A	5316	0	-10.5	NA	unknown	C	C	unknown	PASS	no	unknown	NA	NA	2:8779146
2:8919297	rs116786373	dbSNP_132	A>G	G=4/A=8188	G=139/A=3527	G=143/A=11715	0.0488/3.7916/1.2059	GG=0/GA=4/AA=4092	GG=2/GA=135/AA=1696	GG=2/GA=139/AA=5788	54	KIDINS220	NM_020738.2	intron	NA	c.2371-28T>C	5316	0	-4.48	NA	unknown	A	A	unknown	PASS	no	unknown	NA	NA	2:8779167
2:8919767	rs374695183	dbSNP_138	C>T	T=0/C=8260	T=1/C=3859	T=1/C=12119	0.0/0.0259/0.0083	TT=0/TC=0/CC=4130	TT=0/TC=1/CC=1929	TT=0/TC=1/CC=6059	45	KIDINS220	NM_020738.2	intron	NA	c.2370+37G>A	5316	0	-8.73	NA	unknown	C	C	unknown	PASS	no	unknown	NA	NA	2:8779637
2:8919793	rs376361503	dbSNP_138	C>T	T=1/C=8297	T=0/C=3956	T=1/C=12253	0.0121/0.0/0.0082	TT=0/TC=1/CC=4148	TT=0/TC=0/CC=1978	TT=0/TC=1/CC=6126	62	KIDINS220	NM_020738.2	intron	NA	c.2370+11G>A	5316	0	-2.52	NA	unknown	C	C	unknown	PASS	no	unknown	NA	NA	2:8779663
2:8919823	rs370753728	dbSNP_138	A>C	C=1/A=8321	C=0/A=3980	C=1/A=12301	0.012/0.0/0.0081	CC=0/CA=1/AA=4160	CC=0/CA=0/AA=1990	CC=0/CA=1/AA=6150	90	KIDINS220	NM_020738.2	missense	p.(V784G)	c.2351T>G	5316	0.898	5.3	109	probably-damaging:1.0	A	A	unknown	PASS	no	unknown	NA	NA	2:8779693
2:8919826	rs374203032	dbSNP_138	T>C	C=1/T=8319	C=0/T=3982	C=1/T=12301	0.012/0.0/0.0081	CC=0/CT=1/TT=4159	CC=0/CT=0/TT=1991	CC=0/CT=1/TT=6150	90	KIDINS220	NM_020738.2	missense	p.(K783R)	c.2348A>G	5316	0.987	2.89	26	probably-damaging:0.972	T	T	unknown	PASS	no	unknown	NA	NA	2:8779696
2:8919854	rs368374957	dbSNP_138	C>T	T=1/C=8321	T=0/C=3992	T=1/C=12313	0.012/0.0/0.0081	TT=0/TC=1/CC=4160	TT=0/TC=0/CC=1996	TT=0/TC=1/CC=6156	97	KIDINS220	NM_020738.2	missense	p.(D774N)	c.2320G>A	5316	0.687	5.41	23	probably-damaging:1.0	C	C	unknown	PASS	no	unknown	NA	NA	2:8779724
2:8919855	rs372177879	dbSNP_138	G>A	A=3/G=8317	A=0/G=3974	A=3/G=12291	0.0361/0.0/0.0244	AA=0/AG=3/GG=4157	AA=0/AG=0/GG=1987	AA=0/AG=3/GG=6144	99	KIDINS220	NM_020738.2	coding-synonymous	p.(I773=)	c.2319C>T	5316	0.629	-6.71	NA	unknown	G	G	unknown	PASS	no	unknown	NA	NA	2:8779725
2:8919866	rs374637529	dbSNP_138	C>T	T=1/C=8311	T=0/C=3956	T=1/C=12267	0.012/0.0/0.0082	TT=0/TC=1/CC=4155	TT=0/TC=0/CC=1978	TT=0/TC=1/CC=6133	105	KIDINS220	NM_020738.2	missense	p.(V770M)	c.2308G>A	5316	1	5.41	21	probably-damaging:1.0	C	C	unknown	PASS	no	unknown	NA	NA	2:8779736
2:8919984	rs367675645	dbSNP_138	A>G	G=1/A=8213	G=0/A=3764	G=1/A=11977	0.0122/0.0/0.0083	GG=0/GA=1/AA=4106	GG=0/GA=0/AA=1882	GG=0/GA=1/AA=5988	62	KIDINS220	NM_020738.2	intron	NA	c.2230-40T>C	5316	0.001	0.49	NA	unknown	A	A	unknown	PASS	no	unknown	NA	NA	2:8779854
2:8925819	rs371276084	dbSNP_138	A>G	G=1/A=3981	G=0/A=1752	G=1/A=5733	0.0251/0.0/0.0174	GG=0/GA=1/AA=1990	GG=0/GA=0/AA=876	GG=0/GA=1/AA=2866	54	KIDINS220	NM_020738.2	intron	NA	c.2229+52T>C	5316	0	-0.81	NA	unknown	A	A	unknown	PASS	no	unknown	NA	NA	2:8785689
2:8925883	rs114217936	dbSNP_132	T>C	C=0/T=8258	C=76/T=3732	C=76/T=11990	0.0/1.9958/0.6299	CC=0/CT=0/TT=4129	CC=1/CT=74/TT=1829	CC=1/CT=74/TT=5958	104	KIDINS220	NM_020738.2	coding-synonymous	p.(E739=)	c.2217A>G	5316	1	4.2	NA	unknown	T	T	unknown	PASS	no	unknown	NA	NA	2:8785753
2:8925915	rs200846235	dbSNP_137	C>T	T=1/C=8273	T=0/C=3856	T=1/C=12129	0.0121/0.0/0.0082	TT=0/TC=1/CC=4136	TT=0/TC=0/CC=1928	TT=0/TC=1/CC=6064	115	KIDINS220	NM_020738.2	missense	p.(A729T)	c.2185G>A	5316	0.917	5.37	58	probably-damaging:0.997	C	C	unknown	PASS	yes	unknown	NA	NA	2:8785785
2:8925927	rs372166824	dbSNP_138	G>A	A=1/G=8319	A=0/G=3898	A=1/G=12217	0.012/0.0/0.0082	AA=0/AG=1/GG=4159	AA=0/AG=0/GG=1949	AA=0/AG=1/GG=6108	118	KIDINS220	NM_020738.2	missense	p.(R725C)	c.2173C>T	5316	1	5.37	180	probably-damaging:1.0	G	G	unknown	PASS	no	unknown	NA	NA	2:8785797
2:8925975	rs377048648	dbSNP_138	G>A	A=1/G=8399	A=0/G=4040	A=1/G=12439	0.0119/0.0/0.008	AA=0/AG=1/GG=4199	AA=0/AG=0/GG=2020	AA=0/AG=1/GG=6219	125	KIDINS220	NM_020738.2	missense	p.(R709C)	c.2125C>T	5316	0.997	5.55	180	probably-damaging:0.992	G	G	unknown	PASS	no	unknown	NA	NA	2:8785845
2:8926100	rs139252608	dbSNP_134	A>G	G=0/A=8194	G=1/A=3707	G=1/A=11901	0.0/0.027/0.0084	GG=0/GA=0/AA=4097	GG=0/GA=1/AA=1853	GG=0/GA=1/AA=5950	101	KIDINS220	NM_020738.2	missense	p.(I667T)	c.2000T>C	5316	0.982	5.38	89	benign:0.005	A	A	unknown	PASS	yes	unknown	NA	NA	2:8785970
2:8926126	rs374443314	dbSNP_138	T>C	C=0/T=8180	C=1/T=3699	C=1/T=11879	0.0/0.027/0.0084	CC=0/CT=0/TT=4090	CC=0/CT=1/TT=1849	CC=0/CT=1/TT=5939	104	KIDINS220	NM_020738.2	coding-synonymous	p.(P658=)	c.1974A>G	5316	0.968	-1.98	NA	unknown	T	T	unknown	PASS	no	unknown	NA	NA	2:8785996
2:8926131	rs377758428	dbSNP_138	G>A	A=0/G=8178	A=1/G=3691	A=1/G=11869	0.0/0.0271/0.0084	AA=0/AG=0/GG=4089	AA=0/AG=1/GG=1845	AA=0/AG=1/GG=5934	103	KIDINS220	NM_020738.2	missense	p.(L657F)	c.1969C>T	5316	0.878	5.38	22	probably-damaging:0.98	G	G	unknown	PASS	no	unknown	NA	NA	2:8786001
2:8926176	rs370111258	dbSNP_138	T>C	C=0/T=8168	C=1/T=3643	C=1/T=11811	0.0/0.0274/0.0085	CC=0/CT=0/TT=4084	CC=0/CT=1/TT=1821	CC=0/CT=1/TT=5905	59	KIDINS220	NM_020738.2	intron	NA	c.1940-16A>G	5316	0	-5.48	NA	unknown	T	T	unknown	PASS	no	unknown	NA	NA	2:8786046
2:8926202	rs372453243	dbSNP_138	A>T	T=1/A=8177	T=0/A=3668	T=1/A=11845	0.0122/0.0/0.0084	TT=0/TA=1/AA=4088	TT=0/TA=0/AA=1834	TT=0/TA=1/AA=5922	46	KIDINS220	NM_020738.2	intron	NA	c.1940-42T>A	5316	0.001	-0.19	NA	unknown	A	A	unknown	PASS	no	unknown	NA	NA	2:8786072
2:8926304	rs377033539	dbSNP_138	T>C	C=1/T=8229	C=0/T=3734	C=1/T=11963	0.0122/0.0/0.0084	CC=0/CT=1/TT=4114	CC=0/CT=0/TT=1867	CC=0/CT=1/TT=5981	69	KIDINS220	NM_020738.2	intron	NA	c.1939+32A>G	5316	0	-7.74	NA	unknown	T	T	unknown	PASS	no	unknown	NA	NA	2:8786174
2:8926417	rs370400615	dbSNP_138	T>C	C=2/T=8172	C=0/T=3696	C=2/T=11868	0.0245/0.0/0.0168	CC=0/CT=2/TT=4085	CC=0/CT=0/TT=1848	CC=0/CT=2/TT=5933	158	KIDINS220	NM_020738.2	missense	p.(T620A)	c.1858A>G	5316	0.937	6.07	58	probably-damaging:0.999	T	T	unknown	PASS	no	unknown	NA	NA	2:8786287
2:8928758	rs374089195	dbSNP_138	G>T	T=1/G=8181	T=0/G=3670	T=1/G=11851	0.0122/0.0/0.0084	TT=0/TG=1/GG=4090	TT=0/TG=0/GG=1835	TT=0/TG=1/GG=5925	94	KIDINS220	NM_020738.2	intron	NA	c.1787+19C>A	5316	0.004	1.47	NA	unknown	G	G	unknown	PASS	no	unknown	NA	NA	2:8788628
2:8928782	rs376216731	dbSNP_138	A>C	C=1/A=8189	C=0/A=3710	C=1/A=11899	0.0122/0.0/0.0084	CC=0/CA=1/AA=4094	CC=0/CA=0/AA=1855	CC=0/CA=1/AA=5949	113	KIDINS220	NM_020738.2	coding-synonymous	p.(P594=)	c.1782T>G	5316	1	2.39	NA	unknown	A	A	unknown	PASS	no	unknown	NA	NA	2:8788652
2:8928827	rs370859519	dbSNP_138	A>G	G=4/A=8212	G=0/A=3762	G=4/A=11974	0.0487/0.0/0.0334	GG=0/GA=4/AA=4104	GG=0/GA=0/AA=1881	GG=0/GA=4/AA=5985	120	KIDINS220	NM_020738.2	coding-synonymous	p.(F579=)	c.1737T>C	5316	0.977	-3.32	NA	unknown	A	A	unknown	PASS	no	unknown	NA	NA	2:8788697
2:8928832	rs374312363	dbSNP_138	T>A	A=0/T=8216	A=1/T=3757	A=1/T=11973	0.0/0.0266/0.0084	AA=0/AT=0/TT=4108	AA=0/AT=1/TT=1878	AA=0/AT=1/TT=5986	121	KIDINS220	NM_020738.2	missense	p.(M578L)	c.1732A>T	5316	1	5.93	15	probably-damaging:0.957	T	T	unknown	PASS	no	unknown	NA	NA	2:8788702
2:8928874	rs181547901	dbSNP_135	A>G	G=1/A=8231	G=1/A=3785	G=2/A=12016	0.0121/0.0264/0.0166	GG=0/GA=1/AA=4115	GG=0/GA=1/AA=1892	GG=0/GA=2/AA=6007	127	KIDINS220	NM_020738.2	coding-synonymous	p.(L564=)	c.1690T>C	5316	0.914	-1.82	NA	unknown	A	A	unknown	PASS	no	unknown	NA	NA	2:8788744
2:8928894	rs201557833	dbSNP_137	G>A	A=0/G=8224	A=18/G=3774	A=18/G=11998	0.0/0.4747/0.1498	AA=0/AG=0/GG=4112	AA=0/AG=18/GG=1878	AA=0/AG=18/GG=5990	125	KIDINS220	NM_020738.2	missense	p.(A557V)	c.1670C>T	5316	1	5.93	64	probably-damaging:0.999	G	G	unknown	PASS	yes	unknown	NA	NA	2:8788764
2:8928921	rs200581891	dbSNP_137	C>T	T=0/C=8220	T=1/C=3771	T=1/C=11991	0.0/0.0265/0.0083	TT=0/TC=0/CC=4110	TT=0/TC=1/CC=1885	TT=0/TC=1/CC=5995	113	KIDINS220	NM_020738.2	missense	p.(R548Q)	c.1643G>A	5316	0.994	5.93	43	probably-damaging:1.0	C	C	unknown	PASS	no	unknown	NA	NA	2:8788791
2:8929968	rs191002016	dbSNP_135	C>T	T=0/C=8114	T=16/C=3574	T=16/C=11688	0.0/0.4457/0.1367	TT=0/TC=0/CC=4057	TT=0/TC=16/CC=1779	TT=0/TC=16/CC=5836	22	KIDINS220	NM_020738.2	intron	NA	c.1621+42G>A	5316	0.003	-0.17	NA	unknown	C	C	unknown	PASS	no	unknown	NA	NA	2:8789838
2:8929969	rs184097278	dbSNP_135	G>A	A=38/G=8082	A=0/G=3594	A=38/G=11676	0.468/0.0/0.3244	AA=0/AG=38/GG=4022	AA=0/AG=0/GG=1797	AA=0/AG=38/GG=5819	24	KIDINS220	NM_020738.2	intron	NA	c.1621+41C>T	5316	0.004	0.81	NA	unknown	G	G	unknown	PASS	no	unknown	NA	NA	2:8789839
2:8930003	rs376950810	dbSNP_138	G>T	T=1/G=8149	T=0/G=3614	T=1/G=11763	0.0123/0.0/0.0085	TT=0/TG=1/GG=4074	TT=0/TG=0/GG=1807	TT=0/TG=1/GG=5881	41	KIDINS220	NM_020738.2	intron	NA	c.1621+7C>A	5316	0.2	0.08	NA	unknown	G	G	unknown	PASS	no	unknown	NA	NA	2:8789873
2:8930037	rs369544460	dbSNP_138	A>G	G=0/A=8184	G=3/A=3639	G=3/A=11823	0.0/0.0824/0.0254	GG=0/GA=0/AA=4092	GG=0/GA=3/AA=1818	GG=0/GA=3/AA=5910	75	KIDINS220	NM_020738.2	missense	p.(F532L)	c.1594T>C	5316	1	4.15	22	benign:0.0	A	A	unknown	PASS	no	unknown	NA	NA	2:8789907
2:8930069	rs372243506	dbSNP_138	T>G	G=1/T=8189	G=0/T=3676	G=1/T=11865	0.0122/0.0/0.0084	GG=0/GT=1/TT=4094	GG=0/GT=0/TT=1838	GG=0/GT=1/TT=5932	104	KIDINS220	NM_020738.2	missense	p.(H521P)	c.1562A>C	5316	0.08	5.2	77	benign:0.011	T	T	unknown	PASS	no	unknown	NA	NA	2:8789939
2:8930075	rs376485014	dbSNP_138	G>A	A=1/G=8193	A=0/G=3678	A=1/G=11871	0.0122/0.0/0.0084	AA=0/AG=1/GG=4096	AA=0/AG=0/GG=1839	AA=0/AG=1/GG=5935	108	KIDINS220	NM_020738.2	missense	p.(T519M)	c.1556C>T	5316	0.58	4.32	81	probably-damaging:1.0	G	G	unknown	PASS	no	unknown	NA	NA	2:8789945
2:8930128	rs202154759	dbSNP_137	T>C	C=9/T=8191	C=0/T=3690	C=9/T=11881	0.1098/0.0/0.0757	CC=0/CT=9/TT=4091	CC=0/CT=0/TT=1845	CC=0/CT=9/TT=5936	121	KIDINS220	NM_020738.2	missense	p.(I501M)	c.1503A>G	5316	0.001	-8.93	10	possibly-damaging:0.785	T	T	unknown	PASS	yes	unknown	NA	NA	2:8789998
2:8930215	rs187691112	dbSNP_135	T>G	G=0/T=8170	G=2/T=3646	G=2/T=11816	0.0/0.0548/0.0169	GG=0/GT=0/TT=4085	GG=0/GT=2/TT=1822	GG=0/GT=2/TT=5907	55	KIDINS220	NM_020738.2	intron	NA	c.1442-26A>C	5316	0	-8.2	NA	unknown	T	T	unknown	PASS	no	unknown	NA	NA	2:8790085
2:8931143	rs201844046	dbSNP_137	A>G	G=0/A=8156	G=1/A=3627	G=1/A=11783	0.0/0.0276/0.0085	GG=0/GA=0/AA=4078	GG=0/GA=1/AA=1813	GG=0/GA=1/AA=5891	36	KIDINS220	NM_020738.2	intron	NA	c.1441+47T>C	5316	0.085	1.66	NA	unknown	A	A	unknown	PASS	no	unknown	NA	NA	2:8791013
2:8931212	rs369237426	dbSNP_138	A>G	G=1/A=8191	G=0/A=3738	G=1/A=11929	0.0122/0.0/0.0084	GG=0/GA=1/AA=4095	GG=0/GA=0/AA=1869	GG=0/GA=1/AA=5964	92	KIDINS220	NM_020738.2	coding-synonymous	p.(S473=)	c.1419T>C	5316	0.913	-6.65	NA	unknown	A	A	unknown	PASS	no	unknown	NA	NA	2:8791082
2:8931260	rs372815136	dbSNP_138	C>T	T=1/C=8239	T=0/C=3798	T=1/C=12037	0.0121/0.0/0.0083	TT=0/TC=1/CC=4119	TT=0/TC=0/CC=1899	TT=0/TC=1/CC=6018	98	KIDINS220	NM_020738.2	coding-synonymous	p.(Q457=)	c.1371G>A	5316	1	3.65	NA	unknown	C	G	unknown	PASS	no	unknown	NA	NA	2:8791130
2:8931339	rs375855694	dbSNP_138	G>A	A=1/G=8233	A=0/G=3794	A=1/G=12027	0.0121/0.0/0.0083	AA=0/AG=1/GG=4116	AA=0/AG=0/GG=1897	AA=0/AG=1/GG=6013	79	KIDINS220	NM_020738.2	missense	p.(T431I)	c.1292C>T	5316	0.882	4.53	89	possibly-damaging:0.917	G	G	unknown	PASS	no	unknown	NA	NA	2:8791209
2:8931377	rs145172075	dbSNP_134	CTTACA>C	A1=136/R=7730	A1=199/R=3419	A1=335/R=11149	1.729/5.5003/2.9171	A1A1=1/A1R=134/RR=3798	A1A1=6/A1R=187/RR=1616	A1A1=7/A1R=321/RR=5414	53	KIDINS220	NM_020738.2	intron	NA	c.1277-28_1277-24del5	5316	0	1.12	unknown	unknown	CTTACA	unknown	unknown	PASS	no	unknown	NA	NA	2:8791247
2:8931390	rs199734152	dbSNP_137	G>C	C=1/G=8207	C=0/G=3740	C=1/G=11947	0.0122/0.0/0.0084	CC=0/CG=1/GG=4103	CC=0/CG=0/GG=1870	CC=0/CG=1/GG=5973	48	KIDINS220	NM_020738.2	intron	NA	c.1277-36C>G	5316	0	-0.83	NA	unknown	G	G	unknown	PASS	no	unknown	NA	NA	2:8791260
2:8931393	rs373048700	dbSNP_138	G>A	A=1/G=8207	A=0/G=3732	A=1/G=11939	0.0122/0.0/0.0084	AA=0/AG=1/GG=4103	AA=0/AG=0/GG=1866	AA=0/AG=1/GG=5969	46	KIDINS220	NM_020738.2	intron	NA	c.1277-39C>T	5316	0.001	1.17	NA	unknown	G	G	unknown	PASS	no	unknown	NA	NA	2:8791263
2:8934090	rs371057322	dbSNP_138	T>C	C=1/T=8153	C=0/T=3644	C=1/T=11797	0.0123/0.0/0.0085	CC=0/CT=1/TT=4076	CC=0/CT=0/TT=1822	CC=0/CT=1/TT=5898	78	KIDINS220	NM_020738.2	missense	p.(I376V)	c.1126A>G	5316	0.891	5.83	29	probably-damaging:0.994	T	unknown	unknown	PASS	no	unknown	NA	NA	2:8793960
2:8934091	rs113991487	dbSNP_132	A>G	G=7/A=8147	G=0/A=3642	G=7/A=11789	0.0858/0.0/0.0593	GG=0/GA=7/AA=4070	GG=0/GA=0/AA=1821	GG=0/GA=7/AA=5891	77	KIDINS220	NM_020738.2	coding-synonymous	p.(A375=)	c.1125T>C	5316	0.865	-4.69	NA	unknown	A	unknown	unknown	PASS	no	unknown	NA	NA	2:8793961
2:8934135	rs189545960	dbSNP_135	G>A	A=1/G=8131	A=2/G=3578	A=3/G=11709	0.0123/0.0559/0.0256	AA=0/AG=1/GG=4065	AA=0/AG=2/GG=1788	AA=0/AG=3/GG=5853	45	KIDINS220	NM_020738.2	intron	NA	c.1099-18C>T	5316	0.004	2.41	NA	unknown	G	unknown	unknown	PASS	no	unknown	NA	NA	2:8794005
2:8934153	unknown	none	TC>T	A1=1/R=7801	A1=0/R=3470	A1=1/R=11271	0.0128/0.0/0.0089	A1A1=0/A1R=1/RR=3900	A1A1=0/A1R=0/RR=1735	A1A1=0/A1R=1/RR=5635	33	KIDINS220	NM_020738.2	intron	NA	c.1099-37del1	5316	0	-3.04	unknown	unknown	TC	unknown	unknown	PASS	no	unknown	NA	NA	2:8794023
2:8936861	rs142392886	dbSNP_134	C>T	T=0/C=8334	T=21/C=3931	T=21/C=12265	0.0/0.5314/0.1709	TT=0/TC=0/CC=4167	TT=0/TC=21/CC=1955	TT=0/TC=21/CC=6122	137	KIDINS220	NM_020738.2	intron	NA	c.1098+40G>A	5316	0	-0.98	NA	unknown	C	C	unknown	PASS	no	unknown	NA	NA	2:8796731
2:8936870	rs374882186	dbSNP_138	A>G	G=3/A=8325	G=1/A=3939	G=4/A=12264	0.036/0.0254/0.0326	GG=0/GA=3/AA=4161	GG=0/GA=1/AA=1969	GG=0/GA=4/AA=6130	150	KIDINS220	NM_020738.2	intron	NA	c.1098+31T>C	5316	0	0.42	NA	unknown	A	A	unknown	PASS	no	unknown	NA	NA	2:8796740
2:8936885	rs367848355	dbSNP_138	C>T	T=1/C=8325	T=0/C=3978	T=1/C=12303	0.012/0.0/0.0081	TT=0/TC=1/CC=4162	TT=0/TC=0/CC=1989	TT=0/TC=1/CC=6151	179	KIDINS220	NM_020738.2	intron	NA	c.1098+16G>A	5316	0	-2.51	NA	unknown	C	C	unknown	PASS	no	unknown	NA	NA	2:8796755
2:8936897	rs372119995	dbSNP_138	T>C	C=2/T=8346	C=0/T=3964	C=2/T=12310	0.024/0.0/0.0162	CC=0/CT=2/TT=4172	CC=0/CT=0/TT=1982	CC=0/CT=2/TT=6154	208	KIDINS220	NM_020738.2	intron	NA	c.1098+4A>G	5316	0.004	1.17	NA	unknown	T	T	unknown	PASS	no	unknown	NA	NA	2:8796767
2:8936931	rs115226053	dbSNP_132	A>G	G=53/A=8287	G=3/A=3951	G=56/A=12238	0.6355/0.0759/0.4555	GG=0/GA=53/AA=4117	GG=0/GA=3/AA=1974	GG=0/GA=56/AA=6091	258	KIDINS220	NM_020738.2	coding-synonymous	p.(D356=)	c.1068T>C	5316	0.964	-1.33	NA	unknown	A	A	unknown	PASS	no	unknown	NA	NA	2:8796801
2:8937011	rs368636078	dbSNP_138	A>C	C=4/A=8288	C=0/A=3868	C=4/A=12156	0.0482/0.0/0.0329	CC=0/CA=4/AA=4142	CC=0/CA=0/AA=1934	CC=0/CA=4/AA=6076	164	KIDINS220	NM_020738.2	intron	NA	c.1000-12T>G	5316	0.002	1.74	NA	unknown	A	A	unknown	PASS	no	unknown	NA	NA	2:8796881
2:8937037	rs372228382	dbSNP_138	C>T	T=0/C=8270	T=2/C=3840	T=2/C=12110	0.0/0.0521/0.0165	TT=0/TC=0/CC=4135	TT=0/TC=2/CC=1919	TT=0/TC=2/CC=6054	122	KIDINS220	NM_020738.2	intron	NA	c.1000-38G>A	5316	0	1.28	NA	unknown	C	C	unknown	PASS	no	unknown	NA	NA	2:8796907
2:8938309	rs376842328	dbSNP_138	C>A	A=0/C=8214	A=1/C=3745	A=1/C=11959	0.0/0.0267/0.0084	AA=0/AC=0/CC=4107	AA=0/AC=1/CC=1872	AA=0/AC=1/CC=5979	93	KIDINS220	NM_020738.2	intron	NA	c.999+23G>T	5316	0.009	0.95	NA	unknown	C	C	unknown	PASS	no	unknown	NA	NA	2:8798179
2:8938322	rs369877582	dbSNP_138	C>T	T=1/C=8203	T=0/C=3764	T=1/C=11967	0.0122/0.0/0.0084	TT=0/TC=1/CC=4101	TT=0/TC=0/CC=1882	TT=0/TC=1/CC=5983	106	KIDINS220	NM_020738.2	intron	NA	c.999+10G>A	5316	0	2.2	NA	unknown	C	C	unknown	PASS	no	unknown	NA	NA	2:8798192
2:8938357	rs77973158	dbSNP_131	T>C	C=4/T=8198	C=1/T=3761	C=5/T=11959	0.0488/0.0266/0.0418	CC=0/CT=4/TT=4097	CC=0/CT=1/TT=1880	CC=0/CT=5/TT=5977	128	KIDINS220	NM_020738.2	missense	p.(N325S)	c.974A>G	5316	0.977	4.26	46	probably-damaging:0.96	T	T	unknown	PASS	yes	unknown	NA	NA	2:8798227
2:8938440	unknown	none	T>TA	A1=4/R=7802	A1=103/R=3391	A1=107/R=11193	0.0512/2.9479/0.9469	A1A1=0/A1R=4/RR=3899	A1A1=2/A1R=99/RR=1646	A1A1=2/A1R=103/RR=5545	74	KIDINS220	NM_020738.2	intron	NA	c.901-11_901-10insT	5316	0	-5	unknown	unknown	T	unknown	unknown	PASS	no	unknown	NA	NA	2:8798310
2:8938458	rs374785116	dbSNP_138	T>G	G=1/T=8139	G=0/T=3620	G=1/T=11759	0.0123/0.0/0.0085	GG=0/GT=1/TT=4069	GG=0/GT=0/TT=1810	GG=0/GT=1/TT=5879	61	KIDINS220	NM_020738.2	intron	NA	c.901-28A>C	5316	0.007	3.52	NA	unknown	T	T	unknown	PASS	no	unknown	NA	NA	2:8798328
2:8938462	rs369133109	dbSNP_138	T>C	C=0/T=8138	C=1/T=3613	C=1/T=11751	0.0/0.0277/0.0085	CC=0/CT=0/TT=4069	CC=0/CT=1/TT=1806	CC=0/CT=1/TT=5875	55	KIDINS220	NM_020738.2	intron	NA	c.901-32A>G	5316	0	-6.71	NA	unknown	T	T	unknown	PASS	no	unknown	NA	NA	2:8798332
2:8940534	rs372946383	dbSNP_138	C>A	A=1/C=8271	A=0/C=3866	A=1/C=12137	0.0121/0.0/0.0082	AA=0/AC=1/CC=4135	AA=0/AC=0/CC=1933	AA=0/AC=1/CC=6068	161	KIDINS220	NM_020738.2	missense	p.(G299V)	c.896G>T	5316	1	5.68	109	probably-damaging:1.0	C	C	unknown	PASS	no	unknown	NA	NA	2:8800404
2:8940547	rs376700633	dbSNP_138	T>C	C=1/T=8275	C=0/T=3846	C=1/T=12121	0.0121/0.0/0.0082	CC=0/CT=1/TT=4137	CC=0/CT=0/TT=1923	CC=0/CT=1/TT=6060	177	KIDINS220	NM_020738.2	missense	p.(I295V)	c.883A>G	5316	1	5.68	29	possibly-damaging:0.712	T	T	unknown	PASS	no	unknown	NA	NA	2:8800417
2:8940548	rs199983082	dbSNP_137	A>G	G=1/A=8273	G=0/A=3844	G=1/A=12117	0.0121/0.0/0.0083	GG=0/GA=1/AA=4136	GG=0/GA=0/AA=1922	GG=0/GA=1/AA=6058	178	KIDINS220	NM_020738.2	coding-synonymous	p.(D294=)	c.882T>C	5316	0.998	0.62	NA	unknown	A	A	unknown	PASS	no	unknown	NA	NA	2:8800418
2:8940573	rs373394276	dbSNP_138	C>T	T=1/C=8261	T=0/C=3836	T=1/C=12097	0.0121/0.0/0.0083	TT=0/TC=1/CC=4130	TT=0/TC=0/CC=1918	TT=0/TC=1/CC=6048	195	KIDINS220	NM_020738.2	missense	p.(R286Q)	c.857G>A	5316	1	5.68	43	probably-damaging:1.0	C	C	unknown	PASS	no	unknown	NA	NA	2:8800443
2:8943033	rs186832806	dbSNP_135	G>T	T=1/G=8291	T=0/G=3922	T=1/G=12213	0.0121/0.0/0.0082	TT=0/TG=1/GG=4145	TT=0/TG=0/GG=1961	TT=0/TG=1/GG=6106	64	KIDINS220	NM_020738.2	intron	NA	c.801+27C>A	5316	0	-2.47	NA	unknown	G	G	unknown	PASS	no	unknown	NA	NA	2:8802903
2:8943043	rs115574143	dbSNP_132	T>A	A=0/T=8316	A=64/T=3860	A=64/T=12176	0.0/1.631/0.5229	AA=0/AT=0/TT=4158	AA=1/AT=62/TT=1899	AA=1/AT=62/TT=6057	71	KIDINS220	NM_020738.2	intron	NA	c.801+17A>T	5316	0	-1.51	NA	unknown	T	T	unknown	PASS	no	unknown	NA	NA	2:8802913
2:8943051	rs374732444	dbSNP_138	A>G	G=1/A=8329	G=0/A=3962	G=1/A=12291	0.012/0.0/0.0081	GG=0/GA=1/AA=4164	GG=0/GA=0/AA=1981	GG=0/GA=1/AA=6145	79	KIDINS220	NM_020738.2	intron	NA	c.801+9T>C	5316	0	-3.72	NA	unknown	A	A	unknown	PASS	no	unknown	NA	NA	2:8802921
2:8943092	rs367738361	dbSNP_138	C>T	T=1/C=8359	T=0/C=4014	T=1/C=12373	0.012/0.0/0.0081	TT=0/TC=1/CC=4179	TT=0/TC=0/CC=2007	TT=0/TC=1/CC=6186	121	KIDINS220	NM_020738.2	missense	p.(D257N)	c.769G>A	5316	0.093	4.67	23	probably-damaging:1.0	C	C	unknown	PASS	no	unknown	NA	NA	2:8802962
2:8943093	rs370591101	dbSNP_138	G>A	A=2/G=8358	A=0/G=4016	A=2/G=12374	0.0239/0.0/0.0162	AA=0/AG=2/GG=4178	AA=0/AG=0/GG=2008	AA=0/AG=2/GG=6186	126	KIDINS220	NM_020738.2	coding-synonymous	p.(L256=)	c.768C>T	5316	0.001	-11.1	NA	unknown	G	G	unknown	PASS	no	unknown	NA	NA	2:8802963
2:8943099	rs374871442	dbSNP_138	A>G	G=1/A=8365	G=0/A=4022	G=1/A=12387	0.012/0.0/0.0081	GG=0/GA=1/AA=4182	GG=0/GA=0/AA=2011	GG=0/GA=1/AA=6193	131	KIDINS220	NM_020738.2	coding-synonymous	p.(D254=)	c.762T>C	5316	0.968	-0.85	NA	unknown	A	A	unknown	PASS	no	unknown	NA	NA	2:8802969
2:8943156	rs368353935	dbSNP_138	A>G	G=1/A=8259	G=0/A=3848	G=1/A=12107	0.0121/0.0/0.0083	GG=0/GA=1/AA=4129	GG=0/GA=0/AA=1924	GG=0/GA=1/AA=6053	124	KIDINS220	NM_020738.2	coding-synonymous	p.(D235=)	c.705T>C	5316	0.72	-7.01	NA	unknown	A	A	unknown	PASS	no	unknown	NA	NA	2:8803026
2:8943291	rs2356658	dbSNP_100	G>A	A=8144/G=0	A=3540/G=78	A=11684/G=78	0.0/2.1559/0.6632	AA=4072/AG=0/GG=0	AA=1732/AG=76/GG=1	AA=5804/AG=76/GG=1	32	KIDINS220	NM_020738.2	intron	NA	c.604-34C>T	5316	0	-4.5	NA	unknown	G	A	unknown	PASS	no	unknown	NA	NA	2:8803161
2:8946430	rs375558079	dbSNP_138	T>G	G=1/T=8191	G=0/T=3664	G=1/T=11855	0.0122/0.0/0.0084	GG=0/GT=1/TT=4095	GG=0/GT=0/TT=1832	GG=0/GT=1/TT=5927	199	KIDINS220	NM_020738.2	missense	p.(M192L)	c.574A>C	5316	1	4.99	15	benign:0.359	T	T	unknown	PASS	no	unknown	NA	NA	2:8806300
2:8946444	rs368553342	dbSNP_138	T>C	C=0/T=8188	C=2/T=3658	C=2/T=11846	0.0/0.0546/0.0169	CC=0/CT=0/TT=4094	CC=0/CT=2/TT=1828	CC=0/CT=2/TT=5922	194	KIDINS220	NM_020738.2	missense	p.(K187R)	c.560A>G	5316	0.329	1.25	26	benign:0.001	T	T	unknown	PASS	no	unknown	NA	NA	2:8806314
2:8946473	rs372275689	dbSNP_138	A>T	T=1/A=8169	T=0/A=3618	T=1/A=11787	0.0122/0.0/0.0085	TT=0/TA=1/AA=4084	TT=0/TA=0/AA=1809	TT=0/TA=1/AA=5893	158	KIDINS220	NM_020738.2	coding-synonymous	p.(A177=)	c.531T>A	5316	0.935	-2.11	NA	unknown	A	A	unknown	PASS	no	unknown	NA	NA	2:8806343
2:8946487	rs374716369	dbSNP_138	G>A	A=0/G=8154	A=3/G=3605	A=3/G=11759	0.0/0.0831/0.0255	AA=0/AG=0/GG=4077	AA=0/AG=3/GG=1801	AA=0/AG=3/GG=5878	130	KIDINS220	NM_020738.2	missense	p.(P173S)	c.517C>T	5316	0.995	5.09	74	probably-damaging:1.0	G	G	unknown	PASS	no	unknown	NA	NA	2:8806357
2:8946491	rs369033896	dbSNP_138	G>C	C=1/G=8151	C=0/G=3606	C=1/G=11757	0.0123/0.0/0.0085	CC=0/CG=1/GG=4075	CC=0/CG=0/GG=1803	CC=0/CG=1/GG=5878	121	KIDINS220	NM_020738.2	coding-synonymous	p.(T171=)	c.513C>G	5316	0.97	-3.63	NA	unknown	G	G	unknown	PASS	no	unknown	NA	NA	2:8806361
2:8952480	rs372865465	dbSNP_138	T>C	C=1/T=8221	C=0/T=3792	C=1/T=12013	0.0122/0.0/0.0083	CC=0/CT=1/TT=4110	CC=0/CT=0/TT=1896	CC=0/CT=1/TT=6006	68	KIDINS220	NM_020738.2	intron	NA	c.504+45A>G	5316	0	-4.1	NA	unknown	T	T	unknown	PASS	no	unknown	NA	NA	2:8812350
2:8952536	rs376274980	dbSNP_138	A>T	T=1/A=8297	T=0/A=3928	T=1/A=12225	0.0121/0.0/0.0082	TT=0/TA=1/AA=4148	TT=0/TA=0/AA=1964	TT=0/TA=1/AA=6112	99	KIDINS220	NM_020738.2	missense	p.(C165S)	c.493T>A	5316	1	5.41	112	probably-damaging:1.0	A	A	unknown	PASS	no	unknown	NA	NA	2:8812406
2:8952567	rs200397032	dbSNP_137	A>T	T=1/A=8299	T=0/A=3942	T=1/A=12241	0.012/0.0/0.0082	TT=0/TA=1/AA=4149	TT=0/TA=0/AA=1971	TT=0/TA=1/AA=6120	107	KIDINS220	NM_020738.2	missense	p.(H154Q)	c.462T>A	5316	0.997	2.91	24	benign:0.001	A	A	unknown	PASS	yes	unknown	NA	NA	2:8812437
2:8953328	rs143628210	dbSNP_134	C>T	T=0/C=8166	T=36/C=3616	T=36/C=11782	0.0/0.9858/0.3046	TT=0/TC=0/CC=4083	TT=0/TC=36/CC=1790	TT=0/TC=36/CC=5873	66	KIDINS220	NM_020738.2	intron	NA	c.405+39G>A	5316	0.001	0.42	NA	unknown	C	C	unknown	PASS	no	unknown	NA	NA	2:8813198
2:8953371	rs377662475	dbSNP_138	C>A	A=1/C=8245	A=0/C=3764	A=1/C=12009	0.0121/0.0/0.0083	AA=0/AC=1/CC=4122	AA=0/AC=0/CC=1882	AA=0/AC=1/CC=6004	135	KIDINS220	NM_020738.2	missense	p.(G134V)	c.401G>T	5316	1	6.06	109	probably-damaging:1.0	C	C	unknown	PASS	no	unknown	NA	NA	2:8813241
2:8953375	rs369795631	dbSNP_138	T>C	C=1/T=8247	C=0/T=3772	C=1/T=12019	0.0121/0.0/0.0083	CC=0/CT=1/TT=4123	CC=0/CT=0/TT=1886	CC=0/CT=1/TT=6009	140	KIDINS220	NM_020738.2	missense	p.(T133A)	c.397A>G	5316	1	6.06	58	probably-damaging:0.999	T	T	unknown	PASS	no	unknown	NA	NA	2:8813245
2:8953385	rs374596032	dbSNP_138	A>G	G=2/A=8262	G=0/A=3786	G=2/A=12048	0.0242/0.0/0.0166	GG=0/GA=2/AA=4130	GG=0/GA=0/AA=1893	GG=0/GA=2/AA=6023	150	KIDINS220	NM_020738.2	coding-synonymous	p.(N129=)	c.387T>C	5316	0.923	-4.42	NA	unknown	A	A	unknown	PASS	no	unknown	NA	NA	2:8813255
2:8953411	rs181372484	dbSNP_135	C>G	G=0/C=8286	G=2/C=3792	G=2/C=12078	0.0/0.0527/0.0166	GG=0/GC=0/CC=4143	GG=0/GC=2/CC=1895	GG=0/GC=2/CC=6038	156	KIDINS220	NM_020738.2	missense	p.(E121Q)	c.361G>C	5316	0.014	5.19	29	benign:0.0	C	C	unknown	PASS	yes	unknown	NA	NA	2:8813281
2:8953414	rs199682748	dbSNP_137	C>T	T=4/C=8278	T=1/C=3793	T=5/C=12071	0.0483/0.0264/0.0414	TT=0/TC=4/CC=4137	TT=0/TC=1/CC=1896	TT=0/TC=5/CC=6033	158	KIDINS220	NM_020738.2	missense	p.(V120I)	c.358G>A	5316	0.04	6	29	benign:0.3	C	C	unknown	PASS	no	unknown	NA	NA	2:8813284
2:8953417	rs200899102	dbSNP_137	C>T	T=3/C=8277	T=1/C=3795	T=4/C=12072	0.0362/0.0263/0.0331	TT=0/TC=3/CC=4137	TT=0/TC=1/CC=1897	TT=0/TC=4/CC=6034	160	KIDINS220	NM_020738.2	missense	p.(V119I)	c.355G>A	5316	0.012	5.19	29	probably-damaging:0.999	C	C	unknown	PASS	yes	unknown	NA	NA	2:8813287
2:8953425	rs377100861	dbSNP_138	C>T	T=2/C=8272	T=0/C=3808	T=2/C=12080	0.0242/0.0/0.0166	TT=0/TC=2/CC=4135	TT=0/TC=0/CC=1904	TT=0/TC=2/CC=6039	158	KIDINS220	NM_020738.2	missense	p.(R116H)	c.347G>A	5316	0.966	6.06	29	probably-damaging:0.963	C	C	unknown	PASS	no	unknown	NA	NA	2:8813295
2:8953426	rs370574933	dbSNP_138	G>A	A=0/G=8264	A=3/G=3803	A=3/G=12067	0.0/0.0788/0.0249	AA=0/AG=0/GG=4132	AA=0/AG=3/GG=1900	AA=0/AG=3/GG=6032	155	KIDINS220	NM_020738.2	missense	p.(R116C)	c.346C>T	5316	0.993	4.22	180	benign:0.007	G	G	unknown	PASS	no	unknown	NA	NA	2:8813296
2:8957707	rs184940185	dbSNP_135	T>G	G=1/T=8137	G=20/T=3580	G=21/T=11717	0.0123/0.5556/0.1789	GG=0/GT=1/TT=4068	GG=0/GT=20/TT=1780	GG=0/GT=21/TT=5848	62	KIDINS220	NM_020738.2	intron	NA	c.306+41A>C	5316	0	-3.74	NA	unknown	T	T	unknown	PASS	no	unknown	NA	NA	2:8817577
2:8957755	rs368435770	dbSNP_138	C>T	T=2/C=8206	T=0/C=3750	T=2/C=11956	0.0244/0.0/0.0167	TT=0/TC=2/CC=4102	TT=0/TC=0/CC=1875	TT=0/TC=2/CC=5977	154	KIDINS220	NM_020738.2	missense	p.(R100H)	c.299G>A	5316	0.974	4.71	29	probably-damaging:0.984	C	C	unknown	PASS	no	unknown	NA	NA	2:8817625
2:8957783	rs372167631	dbSNP_138	G>C	C=0/G=8214	C=1/G=3779	C=1/G=11993	0.0/0.0265/0.0083	CC=0/CG=0/GG=4107	CC=0/CG=1/GG=1889	CC=0/CG=1/GG=5996	176	KIDINS220	NM_020738.2	missense	p.(L91V)	c.271C>G	5316	0.179	5.77	32	probably-damaging:0.999	G	G	unknown	PASS	no	unknown	NA	NA	2:8817653
2:8957796	rs189651811	dbSNP_135	G>A	A=0/G=8206	A=2/G=3798	A=2/G=12004	0.0/0.0526/0.0167	AA=0/AG=0/GG=4103	AA=0/AG=2/GG=1898	AA=0/AG=2/GG=6001	178	KIDINS220	NM_020738.2	coding-synonymous	p.(I86=)	c.258C>T	5316	0.031	-4.52	NA	unknown	G	G	unknown	PASS	no	unknown	NA	NA	2:8817666
2:8957831	rs201123643	dbSNP_137	G>A	A=1/G=8187	A=0/G=3774	A=1/G=11961	0.0122/0.0/0.0084	AA=0/AG=1/GG=4093	AA=0/AG=0/GG=1887	AA=0/AG=1/GG=5980	164	KIDINS220	NM_020738.2	missense	p.(L75F)	c.223C>T	5316	0.99	5.77	22	probably-damaging:1.0	G	G	unknown	PASS	no	unknown	NA	NA	2:8817701
2:8957873	rs186236580	dbSNP_135	A>G	G=38/A=8138	G=4/A=3672	G=42/A=11810	0.4648/0.1088/0.3544	GG=0/GA=38/AA=4050	GG=0/GA=4/AA=1834	GG=0/GA=42/AA=5884	82	KIDINS220	NM_020738.2	intron	NA	c.208-27T>C	5316	0.01	2.02	NA	unknown	A	A	unknown	PASS	no	unknown	NA	NA	2:8817743
2:8957897	rs144329227	dbSNP_134	CA>C	A1=0/R=7854	A1=31/R=3539	A1=31/R=11393	0.0/0.8683/0.2714	A1A1=0/A1R=0/RR=3927	A1A1=1/A1R=29/RR=1755	A1A1=1/A1R=29/RR=5682	58	KIDINS220	NM_020738.2	intron	NA	c.208-52del1	5316	0.179	3.82	unknown	unknown	CA	unknown	unknown	PASS	no	unknown	NA	NA	2:8817767
2:8958822	rs374940372	dbSNP_138	T>C	C=1/T=8123	C=0/T=3580	C=1/T=11703	0.0123/0.0/0.0085	CC=0/CT=1/TT=4061	CC=0/CT=0/TT=1790	CC=0/CT=1/TT=5851	88	KIDINS220	NM_020738.2	intron	NA	c.207+3A>G	5316	0.486	3.14	NA	unknown	T	T	unknown	PASS	no	unknown	NA	NA	2:8818692
2:8958858	rs369363850	dbSNP_138	A>C	C=0/A=8162	C=1/A=3643	C=1/A=11805	0.0/0.0274/0.0085	CC=0/CA=0/AA=4081	CC=0/CA=1/AA=1821	CC=0/CA=1/AA=5902	125	KIDINS220	NM_020738.2	missense	p.(I58M)	c.174T>G	5316	0.667	-1.17	10	benign:0.395	A	A	unknown	PASS	no	unknown	NA	NA	2:8818728
2:8958879	rs371742619	dbSNP_138	C>T	T=1/C=8181	T=0/C=3668	T=1/C=11849	0.0122/0.0/0.0084	TT=0/TC=1/CC=4090	TT=0/TC=0/CC=1834	TT=0/TC=1/CC=5924	119	KIDINS220	NM_020738.2	coding-synonymous	p.(L51=)	c.153G>A	5316	1	3.71	NA	unknown	C	C	unknown	PASS	no	unknown	NA	NA	2:8818749
2:8958894	rs377042462	dbSNP_138	G>A	A=1/G=8195	A=0/G=3700	A=1/G=11895	0.0122/0.0/0.0084	AA=0/AG=1/GG=4097	AA=0/AG=0/GG=1850	AA=0/AG=1/GG=5947	103	KIDINS220	NM_020738.2	coding-synonymous	p.(A46=)	c.138C>T	5316	0.325	-5.16	NA	unknown	G	G	unknown	PASS	no	unknown	NA	NA	2:8818764
2:8958918	rs369671667	dbSNP_138	G>C	C=0/G=8210	C=3/G=3715	C=3/G=11925	0.0/0.0807/0.0252	CC=0/CG=0/GG=4105	CC=0/CG=3/GG=1856	CC=0/CG=3/GG=5961	87	KIDINS220	NM_020738.2	coding-synonymous	p.(G38=)	c.114C>G	5316	1	4.7	NA	unknown	G	G	unknown	PASS	no	unknown	NA	NA	2:8818788
2:8958920	rs374455681	dbSNP_138	C>T	T=0/C=8212	T=1/C=3717	T=1/C=11929	0.0/0.0269/0.0084	TT=0/TC=0/CC=4106	TT=0/TC=1/CC=1858	TT=0/TC=1/CC=5964	87	KIDINS220	NM_020738.2	missense	p.(G38S)	c.112G>A	5316	1	5.6	56	probably-damaging:1.0	C	C	unknown	PASS	no	unknown	NA	NA	2:8818790
2:8958927	rs200074000	dbSNP_137	G>C	C=1/G=8209	C=0/G=3714	C=1/G=11923	0.0122/0.0/0.0084	CC=0/CG=1/GG=4104	CC=0/CG=0/GG=1857	CC=0/CG=1/GG=5961	79	KIDINS220	NM_020738.2	intron	NA	c.109-4C>G	5316	0.015	0.43	NA	unknown	G	G	unknown	PASS	no	unknown	NA	NA	2:8818797
2:8958945	rs369910021	dbSNP_138	G>C	C=0/G=8212	C=1/G=3705	C=1/G=11917	0.0/0.027/0.0084	CC=0/CG=0/GG=4106	CC=0/CG=1/GG=1852	CC=0/CG=1/GG=5958	69	KIDINS220	NM_020738.2	intron	NA	c.109-22C>G	5316	0	-2.56	NA	unknown	G	G	unknown	PASS	no	unknown	NA	NA	2:8818815
2:8967071	rs372995976	dbSNP_138	A>G	G=1/A=8179	G=0/A=3672	G=1/A=11851	0.0122/0.0/0.0084	GG=0/GA=1/AA=4089	GG=0/GA=0/AA=1836	GG=0/GA=1/AA=5925	90	KIDINS220	NM_020738.2	intron	NA	c.108+45T>C	5316	0	-2.87	NA	unknown	A	A	unknown	PASS	no	unknown	NA	NA	2:8826941
2:8967100	rs376227376	dbSNP_138	G>A	A=1/G=8179	A=0/G=3676	A=1/G=11855	0.0122/0.0/0.0084	AA=0/AG=1/GG=4089	AA=0/AG=0/GG=1838	AA=0/AG=1/GG=5927	123	KIDINS220	NM_020738.2	intron	NA	c.108+16C>T	5316	0.001	-0.09	NA	unknown	G	G	unknown	PASS	no	unknown	NA	NA	2:8826970
2:8967135	rs111627230	dbSNP_132	T>A	A=0/T=8180	A=3/T=3669	A=3/T=11849	0.0/0.0817/0.0253	AA=0/AT=0/TT=4090	AA=1/AT=1/TT=1834	AA=1/AT=1/TT=5924	140	KIDINS220	NM_020738.2	missense	p.(D30V)	c.89A>T	5316	1	5.19	152	probably-damaging:0.994	T	T	unknown	PASS	no	unknown	NA	NA	2:8827005
2:8967200	rs201261765	dbSNP_137	G>A	A=0/G=8148	A=4/G=3620	A=4/G=11768	0.0/0.1104/0.034	AA=0/AG=0/GG=4074	AA=0/AG=4/GG=1808	AA=0/AG=4/GG=5882	101	KIDINS220	NM_020738.2	coding-synonymous	p.(S8=)	c.24C>T	5316	0.998	2.63	NA	unknown	G	G	unknown	PASS	no	unknown	NA	NA	2:8827070
2:8967201	rs202051983	dbSNP_137	C>T	T=1/C=8147	T=0/C=3624	T=1/C=11771	0.0123/0.0/0.0085	TT=0/TC=1/CC=4073	TT=0/TC=0/CC=1812	TT=0/TC=1/CC=5885	99	KIDINS220	NM_020738.2	missense	p.(S8N)	c.23G>A	5316	1	5.19	46	benign:0.008	C	C	unknown	PASS	no	unknown	NA	NA	2:8827071
2:8967229	rs370863668	dbSNP_138	C>T	T=0/C=8138	T=1/C=3601	T=1/C=11739	0.0/0.0278/0.0085	TT=0/TC=0/CC=4069	TT=0/TC=1/CC=1800	TT=0/TC=1/CC=5869	69	KIDINS220	NM_020738.2	utr-5	NA	c.-6G>A	5316	0.995	5.19	NA	unknown	C	C	unknown	PASS	no	unknown	NA	NA	2:8827099
